# Supplementary material for: Genomic Comparison of Two Species of Samsoniella with Other Genera in the Family Cordycipitaceae
Source: J Fungi (Basel). 2023 Nov 27;9(12):1146. doi: 10.3390/jof9121146 (PMC10744563; doi:10.3390/jof9121146)
Supplement: Supplementary file 1 [file jof-09-01146-s001.zip › manuscript-supplementary data.pdf]

Supplementary materials for

Genomic Comparison of Two Species of *Samsoniella* with Other Genera in the Family Cordycipitaceae

Yingling Lu [1,2,3,†], Zhiqin Wang [1,2,†], Yi Wang [3,*], Yue Chen [1,2], Dexiang Tang [1,2] and Hong Yu [1,2,*]

[1] Yunnan Herbal Laboratory, College of Ecology and Environmental Sciences, Yunnan University, Kunming 650504, China; lyinglingua@163.com (Y.L.); w18314560773@163.com (Z.W.); cy106daytoy@163.com (Y.C.); tangdx1516@163.com (D.T.)

[2] The International Joint Research Center for Sustainable Utilization of Cordyceps Bioresources in China and Southeast Asia, Yunnan University, Kunming 650091, China

[3] Laboratory of Forest Plant Cultivation and Utilization, The Key Laboratory of Rare and Endangered Forest Plants of State Forestry Administration, Yunnan Academy of Forestry and Grassland, Kunming 650201, China

* Correspondence: wangyi@ynlky.org.cn (Y.W.); hongyu@ynu.edu.cn (H.Y.); Tel.: +86-186-8716-3524 (Y.W.); +86-137-0067-6633 (H.Y.)

† These authors contributed equally to this work.

Table S1. Information on twelve species belonging to seven genera of Cordycipitaceae.

Item	Strain	Strain number	Genbank accession					
			Genome	SSU	LSU	TEF	RPB1	RPB2
Samsoniella hepiali ICMM 82-2		ICMM 82-2		MN576738	MN576794	MN576964	MN576854	MN576908
Samsoniella hepiali FENG	FENG		GCA_001455915.2					
Samsoniella yunnanensis YFCC 1527		YFCC 1527		MN576756	MN576812	MN576982	MN576872	MN576926
Akanthomyces lecanii	RCEF 1005	CBS 101247	GCA_001636795.1	AF339604	AF339555	DQ522359	DQ522407	DQ522466
Cordyceps cicadae	CC02	RCEF HP090724-31	GCA_002968875.1	MF416605	MF416552	MF416496	MF416653	MF416447

* Correspondence: wangyi@ynlky.org.cn (Y.W.); hongyu@ynu.edu.cn (H.Y); Tel.: +86-186-8716-3524 (Y.W.); +86-137-0067-6633 (H.Y.)

Cordyceps javanica	IJ1G	CBS 134.22	GCA_006981985.1	MF416610	MF416558	MF416504	MF416661	MF416455
Cordyceps fumosorosea	ARSEF 2679	YFCC 4561	GCA_001636725.1	MN576761	MN576817	MN576987	MN576877	MN576931
Beauveria bassiana	ERL836	YFCC 3369	GCA_010099065.1	MN576768	MN576824	MN576994	MN576884	MN576938
Beauveria pseudobassiana	RGM 2184	YFCC 7120	GCA_022985055.1	MN576772	MN576828	MN576998	MN576888	MN576942
Beauveria brongniartii	RCEF 3172	YFCC 3240	GCA_001636735.1	MN576769	MN576825	MN576995	MN576885	MN576939
Lecanicillium fungicola		CBS 992.69	GCA_900169235.1	KM283768	KM283792	KM283816	-	KM283857
Lecanicillium psalliotae	HWLR35	CBS 532.81	GCA_002796755.1	AF339609	AF339560	EF469067	EF469096	EF469112
Simplicillium aogashimaense	72-15.1	JCM 18167	GCA_012273805.1	LC496889	LC496874	LC496904	-	-
Gamszarea kalimantanensis	FJII-L10-SW-P1		GCA_022813335.1	-	-	-	-	-

Table S2. Genomic assembly and functional annotation of *S. hepiali* ICMM 82-2 and *S. yunnanensis* genome.

Item	Value		Item	Count		Percentage (%)	
	S. hepiali **ICMM 82-2**	***S. yunnanensis*** **YFCC 1527**		***S. hepiali*** **ICMM 82-2**	***S. yunnanensis*** **YFCC 1527**	***S. hepiali*** **ICMM 82-2**	***S. yunnanensis*** **YFCC 1527**
Total length(Mb)	35.92	34.17	NR	9385	10350	99.06	97.64
Max length(bp)	831973	2134020	SwissProt	6812	7189	71.90	67.82
GC content(%)	52.85	53.13	KEGG	3819	3891	40.31	36.71
Total genes number	9474	10,600	GO	6456	6897	68.14	65.07

Total genes length(bp)	15064987	16942441	EggNOg	8697	9357	91.80	88.27
Average gene length(bp)	1590.1	1598.3	P450	9233	10331	97.46	97.46
Gene/Genome(%)	43.3145	50.5	TCDB	1711	1792	18.06	16.91
Contigs	568	275					
Scaffolds	493	216					
Contigs N50	273731	726429					
Scaffolds N50	388857	1059069					
Contigs N90	57092	192491					
Scaffolds N90	83225	267125					

Table S3. Genome assembly integrity assessment

**Property**	**Number**		**Percent (%)**	
	***S. hepiali* ICMM 82-2**	***S. yunnanensis* YFCC 1527**	***S. hepiali* ICMM 82-2**	***S. yunnanensis* YFCC 1527**
Complete BUSCOs	3,804	3,807	99.6%	99.7%
Complete and single-copy BUSCOs	3,792	3,794	99.3%	99.4%
Complete and duplicated BUSCOs	12	13	0.3%	0.3%
Fragmented BUSCOss	3	3	0.1%	0.1%

Missing BUSCOs	10	7	0.3%	0.2%
Total BUSCO groups searched	3,817	3,817	100%	100%

Table S4. Genomes basic characteristics of thirteen studied fungi of Cordycipitaceae.

Item	Contigs	Scaffolds	Total length (Mb)	GC content (%)	Scaffold N50 (bp)	Contig N50 (bp)
S. hepiali ICMM 82-2	568	493	35.92	52.85	388857	273731
S. hepiali FENG	287	129	34.68	53.90	2347338	474778
S. yunnanensis YFCC 1527	275	216	34.17	53.13	1059069	726429
A. lecanii	197	130	35.59	53.10	3946369	782161
C. cicadae	1799	595	34.11	52.70	212207	47316
C. javanica	429	102	34.93	53.10	1814181	159243
C. fumosorosea	685	430	33.49	53.60	872179	201388
B. bassiana	15	15	35.48	49.38	3988868	3988868
B. pseudobassiana	45	45	34.57	51.70	3864399	3864399
B. brongniartii	967	241	32.52	51.50	773364	83189
L. fungicola	1073	781	44.57	49.90	154124	123872
L. psalliotae	1267	194	36.13	52.70	2330369	119947
Simplicillium aogashimaense	44	22	29.25	49.00	3194137	1588579

G. kalimantanensis	198	157	30.43	50.50	461914	354034

Table S5. Putative biosynthetic gene clusters (BGCs) coding for secondary metabolites of fourteen species of Cordycipitaceae.

**Species**	**BGCs**	**Location**	**From**	**To**	**Domain**	**Type**	**Most similar known cluster (%)**
S. hepiali ICMM 82-2	Region 2.2	Scaffold2	846359	891194		NRPS	Unknown
	Region 9.1	Scaffold9.g20	69281	72526	A	NRPS	Unknown
	Region 9.2	Scaffold9.g71	235075	249599	P-A-C-P-C-P-C	NRPS	Unknown
	Region 9.3	Scaffold9	635560	689206		NRPS	Unknown
	Region 15.3	Scaffold15.g82	294285	304541	A-C-P-C-P-C	NRPS	Unknown
	Region 17.2	Scaffold17.g140	525976	539394	A-P-A-C-A-C	NRPS	Leucinostatin A/B (10%)
	Region 18.1	Scaffold18.g122	401980	406347	A-C	NRPS	Unknown
	Region 21.2	Scaffold21.g126	407126	409486	A-P-C	NRPS	Unknown
	Region 25.1	Scaffold25.g55	201262	206688	A-P-C-P-C	NRPS	Dimethylcoprogen (100%)
	Region 32.1	Scaffold32.g22	75986	80245	A-C	NRPS	Unknown
	Region 32.2	Scaffold32.g35	114649	117966	A-C	NRPS	Unknown
	Region 37.1	Scaffold37.g35	112246	116328	A-P-T	NRPS	Unknown
	Region 42.1	Scaffold42.g30	94783	109566	A-P-C-A-P-Te	NRPS	Beauvericin (30%)
	Region 42.2	Scaffold42.g47	154526	163022	C-A-P-C-MT-P-C	NRPS	Beauvericin (50%)
	Region 53.1	Scaffold53.g47	140329	144198	A-P-Te-Te	NRPS	Unknown
	Region 64.2	Scaffold64.g22	74596	79278	A-C	NRPS	Unknown
	Region 102.1	Scaffold102.g5	24016	31799	C-A-A-P-Te	NRPS	Unknown
	Region 102.2	Scaffold102.g9	40704	47636	A-A-P-C	NRPS	Unknown
	Region 243.1	Scaffold243.g2	2465	5639	A-P-Te	NRPS	Unknown
	Region 13.3	Scaffold13	492160	534523		NRPS-like	Unknown
	Region 2.1	Scaffold2.g62	224743	233054	KS-AT-DH-MT-ER-KR-ACP	HR-PKS	Carrimycin (11%)

Region 8.1	Scaffold8.g89	292178	299306	KS-AT-DH-ER-KR-ACP	HR-PKS	Unknown
Region 11.1	Scaffold11.g5	17400	25091	KS-AT-DH-MT-ER-KR-ACP	HR-PKS	Unknown
Region 17.1	Scaffold17.g49	210423	217993	KS-AT-DH-ER-KR	HR-PKS	Unknown
Region 41.1	Scaffold41.g75	226218	233870	KS-AT-DH-ER-KR-ACP	HR-PKS	Unknown
Region 57.1	Scaffold57.g17	64522	72072	KS-AT-MT-ER-KR-ACP	HR-PKS	Unknown
Region 4.4	Scaffold4.g226	824577	832718	KS-AT-DH-MT-KR-ACP-Te	PR-PKS	Fujikurin A/B/C/D (50%)
Region 15.2	Scaffold15.g3	10585	15893	AT-DH-MT-KR	PR-PKS	Equisetin (45%)
Region 64.1	Scaffold64.g18	60163	67541	KS-AT-DH-KR-ACP	PR-PKS	Unknown
Region 1.1	Scaffold1.g310	951239	956986	SAT-KS-AT-PT-ACP-ACP	NR-PKS	Ankaflavin/monascin/rubropunctatine/ monascorubrin (8%)
Region 4.1	Scaffold4.g125	482925	484314	AT	NR-PKS	Unknown
Region 4.3	Scaffold4.g188	697395	701631	KS-AT-DH	NR-PKS	Chondrochloren A (11%)
Region 14.1	Scaffold14.g9	25061	25689	KS-AT	NR-PKS	Unknown
Region 15.1	Scaffold15.g2	8447	9630	KS-AT	NR-PKS	Unknown
Region 29.1	Scaffold29.g4	19947	21291	KS	NR-PKS	Unknown
Region 30.2	Scaffold30.g109	335591	342224	SAT-KS-AT-PT-ACP-ACP-Te	NR-PKS	1,,3,6,8-tetrahydroxynaphthalene (100%)
Region 30.1	Scaffold30.g27	116956	118235	CHS	T3PKS	Unknown
Region 4.2	Scaffold4.g187	689329	694713	KR-ACP-C-A-Te	NRPS,PKS	Unknown
Region 11.2	Scaffold11.g34	142798	154170	KS-AT-DH-ER-KR-P-C-A-P-Te	NRPS,PKS	67-121C (20%)
Region 13.2	Scaffold13.g140	455813	468492	KS-AT-DH-MT-KR-P-C-A-P-Te	NRPS,PKS	Fumosorinone (83%)
Region 16.1	Scaffold16.g96	319309	331319	KS-AT-DH-MT-KR-P-C-A-	NRPS,PKS	Curvupallide-B (44%)

					P-Te		
	Region 18.2	Scaffold18.g125	415653	427487	KS-AT-DH-MT-KR-P-C-A-Te	NRPS,PKS	Unknown
	Region 21.1	Scaffold21	113347	159013		NRPS,PKS	Unknown
	Region 27.1	Scaffold27.g76	238256	243153	C-A-KS	NRPS,PKS	Unknown
	Region 29.2	Scaffold29.g92	318775	323907	C-A-KS	NRPS,PKS	Unknown
	Region 89.1	Scaffold89.g4	16190	28270	KS-AT-DH-MT-KR-P-C-A-Te	NRPS,PKS	Ucs1025a (15%)
	Region 3.1	Scaffold3.g131	464036	467946	A-P-Lys2b	NRPS,Other	Unknown
	Region 35.1	Scaffold35.g100	282872	286582	CaiC-C	NRPS,Other	Unknown
	Region 12.1	Scaffold12.g77	582582	588893	FAS_N-SAT-DUF1729-FAS_MaoC-AT	PKS,Other	Unknown
	Region 3.2	Scaffold3	650013	671935		Terpene	Unknown
	Region 7.1	Scaffold7	494371	515831		Terpene	Squalestatin S1 (40%)
	Region 40.1	Scaffold40	14273	135616		Terpene	Unknown
	Region 13.1	Scaffold13.g118	375317	380197	DIT1-CaiC-P-Transferase	Other	Unknown
	Region 40.2	Scaffold40.g56	195435	196358	Te	Other	Unknown
S. hepiali FENG	Region 1.3	Scaffold1.g815	2668981	2673348	A-C	NRPS	Unknown
	Region 3.3	Scaffold3.g714	2233268	2236360	A-P-C	NRPS	Ankaflavin/monascin/rubropunctatine/monascorubrin (8%)
	Region 5.1	Scaffold5.g3	2983	10600	C-A-P-C-A-Te	NRPS	Unknown
	Region 6.1	Scaffold6	13563	56224		NRPS	Unknown
	Region 6.2	Scaffold6.g615	1958978	1964404	A-P-C-P-C	NRPS	Dimethylcoprogen (100%)
	Region 7.3	Scaffold7.g228	719291	727435	A-P-C-P-P-C	NRPS	Beauvericin (50%)
	Region 7.4	Scaffold7.g244	772362	787145	A-P-A-P-Te	NRPS	Beauvericin (30%)
	Region 7.5	Scaffold7.g342	1059607	1062069	C-P-C	NRPS	Unknown

Region 8.1	Scaffold8.g38	112062	125558	P-A-C-A-P-C-A-P-Te	NRPS	Unknown
Region 8.2	Scaffold8.g154	531988	546394	P-A-C-P-C-P-C	NRPS	Unknown
Region 8.3	Scaffold8.g322	1092913	1107396	A-P-A-C-P-C-P-C	NRPS	Unknown
Region 8.6	Scaffold8.g206	709283	712502	A	NRPS	Unknown
Region 10.3	Scaffold10.g271	879333	892751	A-P-A-C-A-P	NRPS	Leucinostatin A/B (10%)
Region 11.1	Scaffold11.g55	155044	158124	A-P-Te	NRPS	Unknown
Region 11.4	Scaffold11.g247	725659	729918	A-C	NRPS	Unknown
Region 11.5	Scaffold11.g261	763335	767612	A-C	NRPS	Unknown
Region 13.1	Scaffold13.g59	230584	234666	A-P-T	NRPS	Unknown
Region 13.3	Scaffold13.g121	423712	428394	A-C	NRPS	Unknown
Region 16.1	Scaffold16.g149	451327	454746	C-A-P-Te	NRPS	Unknown
Region 22.1	Scaffold22.g8	36561	44210	C-A-A-P-Te	NRPS	Unknown
Region 22.2	Scaffold22.g12	53235	60167	A-A-P-C	NRPS	Unknown
Region 24.1	Scaffold24.g3	8740	11085	A-P-Te	NRPS	Unknown
Region 8.5	Scaffold8	1779540	1821783		NRPS-like	Unknown
Region 1.4	Scaffold1.g1131	3692250	3699800	KS-AT-MT-ER-KR-ACP	HR-PKS	Unknown
Region 1.6	Scaffold1.g2045	6619336	6628148	KS-AT-DH-MT-ER-KR-ACP	HR-PKS	Unknown
Region 1.9	Scaffold1.g1735	5644110	5651762	KS-AT-DH-ER-KR-ACP	HR-PKS	Unknown
Region 7.6	Scaffold7.g550	1701786	1710097	KS-AT-DH-MT-ER-KR-ACP	HR-PKS	Pladienolide B (25%)
Region 9.1	Scaffold9.g135	444478	451606	KS-AT-DH-ER-KR-ACP	HR-PKS	Unknown
Region 10.4	Scaffold10.g371	1207977	1217598	KS-AT-DH-ER-KR-ACP-ACP	HR-PKS	Unknown
Region 13.2	Scaffold13.g118	409279	418545	ER-KS-AT-DH-KR-ACP	HR-PKS	Unknown
Region 10.2	Scaffold10.g256	821111	829240	KS-AT-DH-MT-KR-ACP-	PR-PKS	Fujikurin A/B/C/D (50%)

				Te		
Region 44.1	Scaffold44.g4	10830	19079	KS-AT-DH-MT-ER-ACP-ACP	PR-PKS	Pyranonigrin E (100%)
Region 3.2	Scaffold3.g127	389019	393945	KS-AT-DH-MT	NR-PKS	Equisetin (18%)
Region 4.1	Scaffold4.g76	263899	269646	SAT-KS-AT-PT-ACP-ACP	NR-PKS	Unknown
Region 7.1	Scaffold7.g7	18684	25317	SAT-KS-AT-PT-ACP-ACP-Te	NR-PKS	1,3,6,8-tetrahydroxynaphthalene (100%)
Region 7.2	Scaffold7.g79	245082	246361	CHS	T3PKS	Unknown
Region 1.2	Scaffold1.g812	2647841	2659675	KS-AT-DH-MT-KR-ACP-C-A-Te	NRPS,PKS	Unknown
Region 1.5	Scaffold1.g2015	6490633	6502140	KS-AT-DH-ER-KR-P-C-A-Te	NRPS,PKS	6-methylsalicyclic acid (100%)
Region 2.1	Scaffold2.g464	1584787	1590453	A-P-KS-AT-KR-ACP	NRPS,PKS	Unknown
Region 3.1	Scaffold3.g126	381965	387409	KR-P-C-A-Te	NRPS,PKS	Equisetin (18%)
Region 4.2	Scaffold4.g674	2104959	2108828	A-P-Te-KR	NRPS,PKS	Unknown
Region 4.3	Scaffold4.g954	2990796	2995693	C-A-KS	NRPS,PKS	Unknown
Region 4.4	Scaffold4.g1015	3208454	3223278	KS-AT-DH-MT-KR-P-C-A-Te	NRPS,PKS	Wortmanamide A/B (50%)
Region 8.4	Scaffold8.g408	1369856	1381818	KS-AT-DH-MT-KR-P-C-A-P-Te	NRPS,PKS	Equisetin (45%)
Region 9.2	Scaffold9.g286	944957	950772	C-A-KS	NRPS,PKS	Unknown
Region 10.1	Scaffold10.g209	684700	697002	KS-AT-DH-MT-KR-P-C-A-Te	NRPS,PKS	Chondrochloren A (11%)
Region 11.2	Scaffold11.g73	205402	218085	KS-AT-DH-MT-KR-P-C-A-P-Te	NRPS,PKS	Fumosorinone (83%)
Region 14.1	Scaffold14.g143	475646	487761	KS-AT-DH-MT-KR-P-C-A-	NRPS,PKS	Curvupallide-B (44%)

					P-Te		
	Region 2.2	Scaffold2.g659	2193103	2197013	A-P-Lys2b	NRPS,Other	Unknown
	Region 1.8	Scaffold1.g1647	5336828	5347649	KS-AT-DH-MT-ER-KR-Sec	PKS,Other	Unknown
	Region 1.1	Scaffold1	2019855	2041315		Terpene	Squalestatin S1 (40%)
	Region 2.3	Scaffold2	2372331	2394253		Terpene	Clavaric acid (100%)
	Region 13.4	Scaffold13	773147	794490		Terpene	Unknown
	Region 5.2	Scaffold5	200618	222045		indole	Unknown
	Region 1.7	Scaffold1.g1574	5133210	5133974	P	Other	Unknown
	Region 11.3	Scaffold11.g103	295558	300438	DIT1-CaiC-P-Transferase	Other	Unknown
	Region 13.5	Scaffold13.g221	712495	713418	Te	Other	Unknown
S. yunnanensis YFCC 1527	Region 2.1	Scaffold2.g7	18564	24845	P-C-A	NRPS	Unknown
	Region 3.3	Scaffold3.g273	906400	919818	A-P-A-C-A-P-P	NRPS	Leucinostatin A/leucinostatin B (10%)
	Region 5.3	Scaffold5.g368	1168853	1172722	A-P-Te-Te	NRPS	Unknown
	Region 6.3	Scaffold6.g225	723351	731843	C-A-P-C-MT-A-P-P-C	NRPS	Beauvericin (60%)
	Region 6.4	Scaffold6.g241	777077	791863	A-P-A-P-Te	NRPS	Beauvericin (30%)
	Region 6.5	Scaffold6.g332	1058947	1064712	C-A-P-C-P-C	NRPS	Unknown
	Region 8.1	Scaffold8.g125	380740	384788	A-P-C	NRPS	Unknown
	Region 8.2	Scaffold8.g129	394427	408882	C-A-A-A-P-C-A-P-C	NRPS	Unknown
	Region 8.7	Scaffold8.g368	1121229	1125311	A-P-T	NRPS	Unknown
	Region 10.1	Scaffold10.g198	607488	621968	A-P-A-C-P-C-P-C	NRPS	Unknown
	Region 10.2	Scaffold10.g311	999818	1003034	A	NRPS	Unknown
	Region 11.1	Scaffold11.g65	183098	186190	A-P-C	NRPS	Unknown
	Region 11.2	Scaffold11.g256	795017	800443	A-P-C-P-C	NRPS	Dimethylcoprogen (100%)
	Region 18.3	Scaffold18.g161	492492	496751	A-C	NRPS	Unknown
	Region 18.4	Scaffold18.g176	532466	535786	A-C	NRPS	Unknown
	Region 19.2	Scaffold19.g151	527421	531789	A-C	NRPS	Unknown

Region 24.1	Scaffold24.g139	444888	458384	P-A-C-A-P-C-A-P-Te	NRPS	Unknown
Region 30.1	Scaffold30.g88	252874	255171	A-P-Te	NRPS	Unknown
Region 32.2	Scaffold32.g23	74826	77890	A-Te	NRPS	Unknown
Region 33.1	Scaffold33.g72	237127	251531	P-A-C-P-C-P-C	NRPS	Unknown
Region 47.1	Scaffold47.g3	18001	25789	C-A-A-P-C-A-Te	NRPS	Unknown
Region 18.1	Scaffold18	358439	405083		NRPS-like	Unknown
Region 3.1	Scaffold3.g48	143153	155271	KS-AT-DH-MT-KR-P-C-A-P-Te	NRPS,PKS	Curvupallide-B (50%)
Region 3.5	Scaffold3.g327	1091875	1104176	KS-AT-DH-MT-KR-P-C-A-Te	NRPS,PKS	Chondrochloren A (11%)
Region 4.1	Scaffold4.g89	334206	339793	A-P-KS-AT	NRPS,PKS	Unknown
Region 5.1	Scaffold5.g88	296915	301813	C-A-KS	NRPS,PKS	Unknown
Region 19.1	Scaffold19.g148	506281	518115	KS-AT-DH-MT-KR-P-C-A-Te	NRPS,PKS	Unknown
Region 20.2	Scaffold20.g42	167587	179097	KS-AT-DH-ER-KR-P-C-A-P-Te	NRPS,PKS	6-methylsalicyclic acid (100%)
Region 22.1	Scaffold22.g73	246604	258583	KS-AT-DH-MT-KR-P-C-A-P-Te	NRPS,PKS	Equisetin (18%)
Region 32.1	Scaffold32.g7	15680	28338	KS-AT-DH-MT-KR-P-C-A-P-Te	NRPS,PKS	Fumosorinone (83%)
Region 4.2	Scaffold4.g277	927080	930997	A-P-Lys2b	NRPS,Other	Unknown
Region 7.2	Scaffold7.g155	504248	509679	C-A-KS	NRPS,Other	Unknown
Region 8.5	Scaffold8.g305	917255	924539	A-C-UbiH	NRPS,Other	Unknown
Region 16.2	Scaffold16.g177	608288	611998	CaiC-C	NRPS,Other	Unknown
Region 59.1	Scaffold59.g1	429	15429	MFS-A-P-C-P-A-MT	NRPS,Other	AbT1 (100%)
Region 38.1	Scaffold38.g8	18310	29132	KS-AT-DH-MT-ER-KR-	PKS,Other	Unknown

				Sec34		
Region 1.1	Scaffold1.g175	535459	543557	KS-AT-DH-MT-ER-KR-ACP	HR-PKS	Unknown
Region 1.2	Scaffold1.g255	795405	802955	KS-AT-MT-ER-KR-ACP	HR-PKS	Unknown
Region 3.2	Scaffold3.g177	590853	598496	KS-AT-DH-ER-KR-ACP	HR-PKS	Unknown
Region 7.3	Scaffold7.g303	1002081	1009137	KS-AT-DH-ER-KR-ACP	HR-PKS	Unknown
Region 16.1	Scaffold16.g101	349813	358125	KS-AT-DH-MT-ER-KR-ACP	HR-PKS	Phenalamide (50%)
Region 20.1	Scaffold20.g12	41951	50763	KS-AT-DH-MT-ER-KR-ACP	HR-PKS	Unknown
Region 28.1	Scaffold28.g124	370000	377667	KS-AT-DH-ER-KR-ACP	HR-PKS	Unknown
Region 3.4	Scaffold3.g284	961428	969614	KS-AT-DH-MT-KR-ACP-Te	PR-PKS	Fujikurin A/fujikurin B/fujikurin C/fujikurin D (66%)
Region 8.6	Scaffold8.g309	931528	938906	KS-AT-DH-KR-ACP	PR-PKS	Unknown
Region 18.2	Scaffold18.g127	378439	385083	KS-AT-DH-MT-KR-ACP	PR-PKS	Unknown
Region 3.6	Scaffold3.g394	1309390	1310779	AT	NR-PKS	Unknown
Region 6.1	Scaffold6.g12	37102	43775	SAT-KS-AT-PT-ACP-ACP-Te	NR-PKS	1,3,6,8-tetrahydroxynaphthalene (100%)
Region 7.1	Scaffold7.g69	207271	208616	KS	NR-PKS	Unknown
Region 21.2	Scaffold21.g152	458851	461677	AT-ACP-Aes	NR-PKS	Unknown
Region 25.1	Scaffold25.g8	21487	27228	SAT-KS-PT-ACP-ACP	NR-PKS	Ankaflavin/monascin/rubropunctatine/monascorubrin (8%)
Region 6.2	Scaffold6.g84	256679	258351	CHS	T3PKS	Unknown
Region 2.2	Scaffold2	2045788	2067248		Terpene	Squalestatin S1 (40%)
Region 4.3	Scaffold4	1111814	1133736		Terpene	Clavaric acid (100%)
Region 5.2	Scaffold5.g362	1149455	1151134	Terpene_syn_C_2	Terpene	Unknown

	Region 8.3	Scaffold8	554859	576199		Terpene	Unknown
	Region 21.1	Scaffold21	404300	425553		indole	Unknown
	Region 8.4	Scaffold8.g203	635600	636493	Te	Other	Unknown
A. lecanii	Region 1.3	Scaffold1.g704	2352021	2356422	A-C	NRPS	Unknown
	Region 1.12	Scaffold1.g1896	6180718	6195504	C-A-A-P-Te	NRPS	Beauvericin (30%)
	Region 2.4	Scaffold2.g1139	3744918	3750344	A-P-C-P-C	NRPS	Dimethylcoprogen (100%)
	Region 3.1	Scaffold3.g416	1406251	1420311	A-A-A-P-P-C	NRPS	Epichloenin A (100%)
	Region 3.3	Scaffold3.g782	2630514	2633447	C-A	NRPS	Nivalenol/deoxynivalenol/3-acetyldexynivalenol/15-acetyldeoxynivalenol/neosolaniol/calonectrin/apotrichodiol/isotrichotriol/15-decalonectrin/T-2 toxin/3-acetyl T-2 toxin/trichodiene (20%)
	Region 3.7	Scaffold3.g1223	4176845	4180088	A	NRPS	Unknown
	Region 3.8	Scaffold3.g1275	4345436	4359961	P-A-P-C-P-C	NRPS	Unknown
	Region 3.9	Scaffold3.g1408	4838544	4852911	P-A-C-A-P-C-A-Te	NRPS	Unknown
	Region 4.1	Scaffold4.g89	269926	272901	C-P	NRPS	Unknown
	Region 4.2	Scaffold4.g206	648402	652505	A-P-C	NRPS	Unknown
	Region 4.3	Scaffold4.g714	2298795	2302841	A-P-T	NRPS	Unknown
	Region 4.5	Scaffold4.g758	2435366	2440405	C-A-P-C	NRPS	Acetylaranotin (30%)
	Region 5.4	Scaffold5.g622	2002015	2015424	A-P-A-C-A-P-P	NRPS	Leucinostatin A/B (10%)
	Region 6.1	Scaffold6.g294	1021814	1025683	A-P-Te-Te	NRPS	Unknown
	Region 7.2	Scaffold7.g225	701999	706252	A-C	NRPS	Unknown
	Region 8.1	Scaffold8.g1	19099	39073	A-P-A-C	NRPS	Unknown
	Region 8.2	Scaffold8.g389	1274050	1277972	C-A-Te	NRPS	Unknown
	Region 9.1	Scaffold9.g124	374503	383922	C-A-P-C-MT-A-P-P-C	NRPS	Beauvericin (20%)

Region 10.1	Scaffold10.g25	98339	105664	C-A-P-C-A-P-C	NRPS	Trichodiene-11-one (18%)
Region 34.1	Scaffold34.g4	5656	11170	A-C	NRPS	Unknown
Region 70.1	Scaffold70.g1	544	3172	A-P-Te	NRPS	Unknown
Region 7.3	Scaffold 7	1107936	1150983		NRPS-like	Unknown
Region 13.1	Scaffold 13	169882	212101		NRPS-like	Unknown
Region 1.2	Scaffold1.g701	2328461	2340298	KS-AT-DH-MT-KR-P-C-A-P-Te	NRPS,PKS	Unknown
Region 2.1	Scaffold2.g42	153273	164515	KS-AT-DH-ER-KR-P-C-A-P-Te	NRPS,PKS	6-methylsalicyclic acid (100%)
Region 3.5	Scaffold3.g1034	3513244	3519012	C-A-KS	NRPS,PKS	Unknown
Region 5.5	Scaffold5.g956	3107163	3115128	SAT-KS-AT-ACP-ACP-HTH-MT-Aes	NRPS,PKS	Citrinin (12%)
Region 25.1	Scaffold25.g9	21759	33955	KS-AT-DH-MT-KR-P-C-A-P-Te	NRPS,PKS	Aspyridone A (66%)
Region 1.11	Scaffold1.g1873	6105568	6112269	Abhydrolase-WD40-P-C	NRPS,Other	Unknown
Region 2.3	Scaffold2.g401	1349567	1355448	CaiC-C	NRPS,Other	Unknown
Region 5.6	Scaffold5.g978	3171624	3175550	A-Lys2b	NRPS,Other	Unknown
Region 7.1	Scaffold7.g213	670683	677230	MPP-5_nucleotid-C-A-C	NRPS,Other	Unknown
Region 5.1	Scaffold5.g226	706009	708404	CaiC-P-KS	PKS,Other	Unknown
Region 1.6	Scaffold1.g1611	5329545	5337190	KS-AT-DH-ER-KR-ACP	HR-PKS	Unknown
Region 2.5	Scaffold2.g1382	4544923	4553100	KS-AT-DH-MT-ER-KR	HR-PKS	Ajudazol A (46%)
Region 3.2	Scaffold3.g631	2188807	2197541	Te-KS-AT-ER-KR	HR-PKS	Fusaric acid (13%)
Region 3.4	Scaffold3.g866	2900117	2907303	KS-AT-DH-ER-KR-ACP	HR-PKS	Unknown
Region 5.3	Scaffold5.g534	1695716	1703434	KS-AT-DH-ER-KR	HR-PKS	Unknown
Region 2.2	Scaffold2.g86	303850	304632	KS-ACP	NR-PKS	Unknown
Region 3.6	Scaffold3.g1115	3805482	3806825	KS	NR-PKS	Unknown

	Region 3.10	Scaffold3.g1493	5111810	5117568	SAT-KS-AT-PT-ACP	NR-PKS	1,3,6,8-tetrahydroxynaphthalene (100%)
	Region 1.9	Scaffold 1	6005402	6046619		PKS	Unknown
	Region 1.1	Scaffold 1	1718417	1739571		Terpene	Squalestatin S1 (40%)
	Region 2.6	Scaffold 2	4940864	4958923		Terpene	Unknown
	Region 2.7	Scaffold2.g1669	5443697	5447257	Terpene_syn_C_2-MT	Terpene	Unknown
	Region 6.2	Scaffold6.g300	1043765	1045192	Terpene_syn_C_2	Terpene	Unknown
C. cicadae	Region 1.1	Scf00001.g35	119793	123659	A-P-Te	NRPS	Unknown
	Region 1.2	Scf00001.g99	327555	341271	A-A-P-A-P	NRPS	Ferrichrome (66%)
	Region 1.3	Scf00001.g211	685792	690038	A-P-C	NRPS	Unknown
	Region 6.1	Scf00006.g101	377154	381502	A-C	NRPS	Unknown
	Region 38.1	Scf00038.g38	113515	117570	A-P-T	NRPS	Unknown
	Region 39.1	Scf00039.g41	153658	157482	A-P-C	NRPS	Unknown
	Region 39.2	Scf00039.g66	217296	220238	A-P-Te	NRPS	Unknown
	Region 56.1	Scf00056.g6	21350	23149	A	NRPS	Unknown
	Region 56.2	Scf00056.g7	25525	30960	A-P-C-P-C	NRPS	Dimethylcoprogen (100%)
	Region 59.1	Scf00059.g30	88994	91273	A	NRPS	Unknown
	Region 59.2	Scf00059.g35	108003	109628	C	NRPS	Unknown
	Region 87.1	Scf00087.g8	19232	30142	C-A-P-C-A-MT-P-P-C	NRPS	Beauvericin (50%)
	Region 158.1	Scf00158.g12	29605	33008	A-Te	NRPS	Unknown
	Region 183.2	Scf00183.g9	25888	30606	A-C	NRPS	Unknown
	Region 222.1	Scf00222	1	27708		NRPS	Unknown
	Region 53.1	Scf00053.g1	11323	19713	A-HMA-ZntA	NRPS,Other	Unknown
	Region 92.1	Scf00092.g21	58518	63474	DIT-Caic-P-Te	NRPS,Other	Unknown
	Region 6.2	Scf00006.g105	403977	415814	KS-AT-DH-MT-KR-P-C-A-P-Te	NRPS,PKS	Unknown
	Region 27.1	Scf00027.g51	198137	211143	KS-AT-DH-MT-KR-P-C-A-	NRPS,PKS	Wortmanamide A/B (50%)

					P-Te		
	Region 29.1	Scf00029.g74	252612	264375	A-P-KS-AT-P-Te-A-P-C	NRPS,PKS	Unknown
	Region 109.1	Scf00109.g6	9516	21901	KS-AT-DH-MT-KR-P-C-A-Te	NRPS,PKS	Ilicicolin H (33%)
	Region 159.1	Scf00159.g6	12558	24106	KS-AT-DH-ER-KR-P-C-A-P	NRPS,PKS	Unknown
	Region 90.1	Scf00090.g14	49329	56498	KS-AT-DH-ER-KR-ACP	HR-PKS	Unknown
	Region 219.1	Scf00219.g3	12936	20664	KS-AT-DH-MT-ER-KR-ACP	HR-PKS	Leucinostatin A/B (10%)
	Region 183.1	Scf00183.g5	10103	17659	KS-AT-DH-KR-ACP	PR-PKS	Unknown
	Region 12.1	Scf00012.g9	20169	27153	SAT-KS-AT-PT-DH-ACP-ACP-Te	NR-PKS	Neosartorin (15%)
	Region 117.1	Scf00117.g18	49853	57073	SAT-KS-AT-PT-ACP-ACP-ACP-Te	NR-PKS	Viriditoxin (66%)
	Region 40.1	Scf00040	65370	87292		Terpene	Unknown
	Region 51.1	Scf00051	1	18148		Terpene	Unknown
	Region 94.1	Scf00094	2549	23508		Terpene	Squalestatin S1 (40%)
	Region 429.1	Scf00429	1	2570		Indole	Unknown
C. javanica	Region 1.1	Scaffold00001.g2	5759	11206	A-P-C-P-C	NRPS	Dimethylcoprogen (100%)
	Region 1.2	Scaffold00001.g173	590023	596449	A-P-C-A-P-C	NRPS	Unknown
	Region 1.3	Scaffold00001.g191	656454	661042	A-P-C	NRPS	Unknown
	Region 1.5	Scaffold00001.g941	3218740	3233056	P-A-P	NRPS	Emericellamide A/B (60%)
	Region 2.2	Scaffold00002.g680	2431396	2433063	C-A	NRPS	Unknown
	Region 5.1	Scaffold00005.g436	1460417	1466608	P-C-A-C	NRPS	Unknown
	Region 6.2	Scaffold00006.g129	482414	486871	A-C	NRPS	Unknown
	Region 9.1	Scaffold00009.g63	208490	212350	A-P-Te-Te	NRPS	Unknown

Region 10.1	Scaffold00010.g83	319539	335706	P-A-C-P-C-P-C	NRPS	Unknown
Region 10.2	Scaffold00010.g109	421128	434338	A-A-A-P-C-P-C	NRPS	Unknown
Region 10.3	Scaffold00010.g141	528065	531431	A	NRPS	Unknown
Region 11.3	Scaffold00011.g222	707971	712033	A-C	NRPS	Unknown
Region 12.2	Scaffold00012.g257	959060	972490	P-A-A-P	NRPS	Leucinostatin A/B (10%)
Region 14.1	Scaffold00014.g135	485585	489625	A-P-T	NRPS	Unknown
Region 15.1	Scaffold00015.g211	672486	682669	A-P-A-C-A-P-Te	NRPS	Unknown
Region 16.1	Scaffold00016.g92	340796	344287	A-P-Te	NRPS	Unknown
Region 18.2	Scaffold00018.g44	136279	150318	A-A-A-P-P-C	NRPS	Epichloenin A (100%)
Region 18.3	Scaffold00018	206435	250838		NRPS	Unknown
Region 22.1	Scaffold00022.g38	109214	122165	A-P-A-C-A-A-P-C	NRPS	Unknown
Region 11.1	Scaffold00011	230195	272005		NRPS-like	Unknown
Region 23.1	Scaffold00023	169439	211463		NRPS-like	Unknown
Region 2.3	Scaffold00002.g696	2499201	2504987	C-A-KS	NRPS,PKS	Unknown
Region 6.1	Scaffold00006.g126	458187	470027	KS-AT-DH-MT-KR-P-C-A-P-Te	NRPS,PKS	Unknown
Region 7.1	Scaffold00007.g199	692177	704347	KS-AT-DH-MT-KR-P-C-A-P-Te	NRPS,PKS	Fumonisin (11%)
Region 7.2	Scaffold00007.g344	1179642	1191995	KS-AT-MT-KR-P-C-A-Te	NRPS,PKS	Phomasetin (85%)
Region 17.1	Scaffold00017.g157	520093	531601	KS-AT-DH-ER-KR-P-C-P-Te	NRPS,PKS	Unknown
Region 26.1	Scaffold00026.g52	228231	241615	KS-AT-DH-MT-KR-P-C-A-P-Te	NRPS,PKS	Cytochalasin E/K (15%)
Region 4.2	Scaffold00004.g412	1478978	1482822	CaiC-C	NRPS,Other	Unknown
Region 10.4	Scaffold00010.g190	733687	741505	A-P-Lys2b-APH-APH-Prolidase	NRPS,Other	Unknown

Region 11.2	Scaffold00011.g210	676609	683194	MPP-5_nucleotid-C-A-P-C	NRPS,Other	Unknown
Region 39.2	Scaffold00039.g5	18260	28399	A-C-UbiH-CYP	NRPS,Other	Unknown
Region 2.4	Scaffold00002.g784	2821269	2824322	KS-Ubiq_cyt_C_chap	PKS,Other	Unknown
Region 10.5	Scaffold00010.g322	1203435	1219549	KS-AT-MT-P-P-C-A-P-Te-PX-Snx8	NRPS,PKS,Other	Equisetin (18%)
Region 1.4	Scaffold00001.g938	3204176	3212214	KS-AT-DH-MT-ER-KR-ACP	HR-PKS	Emericellamide A/B (60%)
Region 2.1	Scaffold00002.g514	1840786	1848140	KS-AT-DH-ER-KR-ACP	HR-PKS	Unknown
Region 4.1	Scaffold00004.g340	1242746	1250702	KS-AT-DH-ER-KR-ACP	HR-PKS	Lasalocid (12%)
Region 6.5	Scaffold00006.g542	1957031	1965078	KS-AT-DH-MT-ER-KR-ACP	HR-PKS	Pyranonigrin E (100%)
Region 8.1	Scaffold00008.g481	1657613	1665967	KS-AT-DH-MT-ER-KR-ACP	HR-PKS	Mycinamicin II (9%)
Region 11.4	Scaffold00011.g361	1194389	1202199	KS-AT-DH-ER-KR-ACP	HR-PKS	Chaetoviridin E/11-epichaetomugilin A (16%)
Region 12.1	Scaffold00012.g175	650448	657936	KS-AT-DH-ER-KR-ACP	HR-PKS	Unknown
Region 17.2	Scaffold00017.g166	562314	569988	KS-AT-DH-ER-KR-ACP	HR-PKS	Unknown
Region 28.1	Scaffold00028.g57	206133	214183	KS-AT-DH-MT-ER-KR-ACP	HR-PKS	Solanapyrone D (83%)
Region 6.3	Scaffold00006.g258	949011	956664	KS-AT-DH-KR-ACP	PR-PKS	Unknown
Region 39.1	Scaffold00039.g2	4314	11716	KS-AT-KR-ACP	PR-PKS	Unknown
Region 3.1	Scaffold00003.g717	2497670	2506720	SAT-KS-AT-ACP-HTH-MT-Te	NR-PKS	Unknown
Region 11.5	Scaffold00011.g364	1207681	1214331	SAT-KS-AT-PT-ACP-Te	NR-PKS	Chaetoviridin E/11-epichaetomugilin A (16%)
Region 18.1	Scaffold00018.g17	49736	56584	KS-AT-PT-ACP-Te	NR-PKS	Unknown

	Region 22.2	Scaffold00022.g89	309897	315116	SAT-KS-AT-PT-ACP	NR-PKS	Secalonic acids (37%)
	Region 6.4	Scaffold00006	1135006	1156190		Terpene	Squalestatin S1 (40%)
	Region 20.1	Scaffold00020	18222	39457		Terpene	Unknown
	Region 20.2	Scaffold00020.g25	99183	100088	Te	Other	Unknown
	Region 1.1	Scaffold00001.g2	5759	11206	A-P-C-P-C	NRPS	Dimethylcoprogen (100%)
C. fumosorosea	Region 1.1	Scaffold1.g176	566459	575959	C-A-P-C-MT-P-P-C	NRPS	Beauvericin (50%)
	Region 2.2	Scaffold2.g145	502843	516370	A-P-A-C-A-C	NRPS	Leucinostatin A/B (10%)
	Region 2.3	Scaffold2.g211	725196	729062	A-P-Te-Te	NRPS	Unknown
	Region 3.1	Scaffold3.g1	3370	7977	A-P-C-A-P	NRPS	Unknown
	Region 3.2	Scaffold3.g263	859659	861024	A-Te	NRPS	Unknown
	Region 3.3	Scaffold3.g291	922611	926408	A-P-C	NRPS	Unknown
	Region 5.1	Scaffold5.g229	753926	768517	P-A-C-P-C-P-C	NRPS	Unknown
	Region 5.2	Scaffold5.g283	929430	932762	A	NRPS	Unknown
	Region 5.4	Scaffold5.g428	1461007	1464286	A-P	NRPS	Unknown
	Region 6.1	Scaffold6.g45	155767	160163	A-C	NRPS	Unknown
	Region 17.1	Scaffold17.g25	83026	87081	A-P-T	NRPS	Unknown
	Region 18.1	Scaffold18.g43	132342	137714	A-P-C-A-P	NRPS	Unknown
	Region 21.1	Scaffold21.g8	24718	29325	A-P-C-A-P	NRPS	Unknown
	Region 22.1	Scaffold22.g1	227	4642	C-A-P-Te	NRPS	Unknown
	Region 22.2	Scaffold22.g14	58302	59553	A	NRPS	Unknown
	Region 28.1	Scaffold28.g86	262359	266609	A-C	NRPS	Unknown
	Region 32.1	Scaffold32.g52	186177	190784	A-P-C-A-P	NRPS	Unknown
	Region 38.1	Scaffold38.g21	66252	70877	A-C	NRPS	Unknown
	Region 43.2	Scaffold43.g50	127944	131402	A-P-Te	NRPS	Unknown
	Region 72.1	Scaffold72.g8	25723	31524	P-C-A-P	NRPS	Unknown
	Region 75.1	Scaffold75.g2	4056	9497	A-P-C-P-C	NRPS	Dimethylcoprogen (100%)

Region 87.1	Scaffold87.g1	29	5563	A-P-C-A-P	NRPS	Unknown
Region 89.1	Scaffold89.g4	20654	23383	A	NRPS	Unknown
Region 133.1	Scaffold133.g2	2685	7292	A-P-C-A-P	NRPS	Unknown
Region 145.1	Scaffold145.g1	448	5242	A-P-C-A-P	NRPS	Unknown
Region 26.1	Scaffold26	132466	174943		NRPS-like	Unknown
Region 34.3	Scaffold34	65039	108155		NRPS-like	Unknown
Region 43.1	Scaffold43	21531	61753		NRPS-like	Unknown
Region 2.5	Scaffold2.g350	1138065	1150080	A-P-KS-AT-P-Te-A-C	NRPS,PKS	Unknown
Region 5.3	Scaffold5.g340	1134397	1147422	KS-AT-DH-MT-KR-P-C-A-P-Te	NRPS,PKS	Wortmanamide A/B (50%)
Region 6.2	Scaffold6.g49	175727	187558	KS-AT-DH-MT-KR-P-C-A-P-Te	NRPS,PKS	Unknown
Region 15.1	Scaffold15.g147	446839	460663	KS-AT-DH-MT-KR-P-C-A-Te	NRPS,PKS	Fusarin (100%)
Region 20.2	Scaffold20.g170	546631	558137	KS-AT-DH-ER-KR-P-C-A-P-Te	NRPS,PKS	Nivalenol/deoxynivalenol/3-acetyldeoxynivalenol/15-acetyldeoxynivalenol/neosolaniol/calonectrin/apotrichodiol/isotrichotriol/15-decalonectrin/T-2 toxin/3-acetyl T-2 toxin/trichodiene (8%)
Region 31.1	Scaffold31.g65	197423	202323	C-A-KS	NRPS,PKS	Unknown
Region 34.2	Scaffold34.g9	30035	42579	KS-AT-DH-MT-KR-P-C-A-P-Te	NRPS,PKS	Fumosorinone (100%)
Region 4.2	Scaffold4.g442	1473091	1476773	CaiC-C	NRPS,Other	Unknown
Region 34.1	Scaffold34.g5	13338	18624	UbiA-A-P-C-A-P	NRPS,Other	Unknown
Region 123.1	Scaffold123.g2	3637	8957	HET-A-P-C-A-P	NRPS,Other	Unknown

	Region 2.1	Scaffold2.g60	207912	215858	KS-AT-ER-KR-ACP	HR-PKS	Unknown
	Region 7.1	Scaffold7.g116	379879	387019	KS-AT-DH-ER-KR-ACP	HR-PKS	Unknown
	Region 20.1	Scaffold20.g159	510912	518569	KS-AT-DH-ER-KR-ACP	HR-PKS	Unknown
	Region 38.2	Scaffold38.g25	77590	85295	KS-AT-DH-KR-ACP	PR-PKS	Unknown
	Region 1.2	Scaffold1.g294	925998	927220	AT	NR-PKS	Unknown
	Region 1.4	Scaffold1.g781	2500030	2503110	KS	NR-PKS	Unknown
	Region 25.1	Scaffold25.g114	384433	386857	AT	NR-PKS	Unknown
	Region 4.1	Scaffold4.g330	1133764	1140619	SAT-KS-AT-PT-ACP-ACP-Te	NR-PKS	Naphthopyrone (100%)
	Region 58.1	Scaffold58.g11	58616	66054	SAT-KS-AT-PT-ACP-ACP-ACP-Te	NR-PKS	Viriditoxin (22%)
	Region 2.4	Scaffold2	975886	997904		Terpene	Clavaric acid (100%)
	Region 12.1	Scaffold12	1000446	1011099		Terpene	Squalestatin S1 (40%)
	Region 18.3	Scaffold18	370196	391635		Terpene	Unknown
	Region 7.2	Scaffold7	360315	407019		siderophore	Unknown
	Region 1.3	Scaffold1.g586	1870622	1871440	P	Other	Unknown
	Region 18.2	Scaffold18.g97	307055	307903	Te	Other	Unknown
	Region 75.2	Scaffold75.g3	11794	13584	CaiC	Other	Unknown
B. bassiana	Region 1.1	Scaffold1.g157	650718	659950	C-A-C-A-P	NRPS	Unknown
	Region 1.3	Scaffold1.g329	1241870	1245901	A-P-T	NRPS	Unknown
	Region 1.4	Scaffold1.g352	1313745	1320884	C-P-A	NRPS	Unknown
	Region 1.11	Scaffold1.g1633	6258732	6270019	A-Te	NRPS	Unknown
	Region 2.1	Scaffold2.g111	371605	380955	C-A-P-C-MT-P-P-C	NRPS	Beauvericin (90%)
	Region 2.3	Scaffold2.g1348	4941462	4945821	A-C	NRPS	Unknown
	Region 2.5	Scaffold2.g1673	6209121	6210773	P-C	NRPS	Nivalenol/deoxynivalenol/3-acetyldeoxynivalenol/15-

						acetyldeoxynivalenol/neosolaniol/calonectrin/apotrichodiol/isotrichotriol/15-decalonectrin/T-2 toxin/trichodiene (20%)
Region 2.6	Scaffold2.g1675	6214189	6216801	C-A	NRPS	Nivalenol/deoxynivalenol/3-acetyldeoxynivalenol/15-acetyldeoxynivalenol/neosolaniol/calonectrin/apotrichodiol/isotrichotriol/15-decalonectrin/T-2 toxin/trichodiene (20%)
Region 3.4	Scaffold3.g376	1317539	1326979	C-A-P-C-MT-A-P-P-C	NRPS	Bassianolide (86%)
Region 3.6	Scaffold3.g804	3046074	3059672	A-A-P	NRPS	Leucinostatin A/ leucinostatin B (10%)
Region 3.9	Scaffold3.g1093	4115463	4118725	A	NRPS	Unknown
Region 3.10	Scaffold3.g1134	4258477	4273077	P-A-C-P-C-P-C	NRPS	Unknown
Region 4.3	Scaffold4.g835	3124761	3129011	A-C	NRPS	Unknown
Region 5.4	Scaffold5.g401	1450566	1464805	A-P	NRPS	Emericellamide A/ emericellamide B (40%)
Region 5.5	Scaffold5.g982	3389248	3394959	C-A-P-C-P-C	NRPS	Unknown
Region 6.3	Scaffold6.g268	998385	1002962	A-C	NRPS	Unknown
Region 6.6	Scaffold6.g853	3028672	3032541	A-P-Te-Te	NRPS	Unknown
Region 7.1	Scaffold7.g490	1891272	1902068	A-P-A-P-C-P-Te	NRPS	Unknown
Region 8.4	Scaffold8.g318	1455818	1461334	A-P-C-P-C	NRPS	Dimethylcoprogen (100%)
Region 2.2	Scaffold2.g1345	4918823	4930660	KS-AT-DH-MT-KR-P-C-A-P-Te	NRPS,PKS	Unknown
Region 4.4	Scaffold4.g976	3581929	3594921	KS-AT-DH-MT-KR-P-C-A-P-Te	NRPS,PKS	Fumosorinone (83%)

Region 6.5	Scaffold6.g568	2038440	2044184	C-A-P-KS	NRPS,PKS	Unknown
Region 8.7	Scaffold8.g551	2409688	2421685	KS-AT-KR-P-C-A-P-Te	NRPS,PKS	Dihydroisoflavipucine/ isoflavipucine (18%)
Region 1.6	Scaffold1.g1008	3952798	3958207	Sulfotransfer-A-P-C	NRPS,Other	Unknown
Region 3.7	Scaffold3.g942	3566864	3575200	A-P-NAD-Cnl2	NRPS,Other	Unknown
Region 4.5	Scaffold4.g1013	3737811	3742699	DIT1-CaiC-P-Te	NRPS,Other	Unknown
Region 5.1	Scaffold5.g252	972072	977838	CaiC-C-Atrophin	NRPS,Other	Unknown
Region 1.5	Scaffold1.g979	3876790	3885657	KS-AT-DH-MT-ER-KR-ACP	HR-PKS	Unknown
Region 1.8	Scaffold1.g1062	4120532	4129136	KS-AT-DH-MT-ER-KR-ACP	HR-PKS	Leucinostatin A/ leucinostatin B (10%)
Region 1.9	Scaffold1.g1499	5820865	5828601	KS-AT-DH-ER-KR-ACP	HR-PKS	Unknown
Region 3.5	Scaffold3.g730	2739325	2746972	KS-AT-DH-ER-KR-ACP	HR-PKS	Unknown
Region 4.2	Scaffold4.g236	863778	870987	KS-AT-DH-MT-ER-KR-ACP	HR-PKS	leucinostatin A/ leucinostatin B (10%)
Region 5.3	Scaffold5.g398	1435359	1443152	KS-AT-DH-MT-ER-KR-ACP	HR-PKS	Emericellamide A/ emericellamide B (40%)
Region 6.1	Scaffold6.g223	813079	820278	KS-AT-DH-ER-KR-ACP	HR-PKS	Unknown
Region 8.6	Scaffold8.g543	2367411	2374737	KS-AT-ER-KR-ACP	HR-PKS	Dihydroisoflavipucine/ isoflavipucine (18%)
Region 6.2	Scaffold6.g264	983708	990901	KS-AT-DH-KR-ACP	PR-PKS	Unknown
Region 8.5	Scaffold8.g456	1973086	1980424	KS-AT-MT-ER-ACP	PR-PKS	Unknown
Region 3.1	Scaffold3.g84	318691	325487	SAT-KS-AT-PT-ACP-ACP-Te	NR-PKS	neosartorin (10%)
Region 5.2	Scaffold5.g360	1314954	1322210	SAT-KS-AT-PT-ACP-ACP-Te	NR-PKS	Unknown

	Region 6.4	Scaffold6.g480	1746885	1748229	KS	NR-PKS	Unknown
	Region 7.2	Scaffold7.g584	2199410	2200570	AT	NR-PKS	Unknown
	Region 1.7	Scaffold1.g1061	4118849	4128849	CHS	T3PKS	Leucinostatin A/ leucinostatin B (10%)
	Region 1.10	Scaffold1.g1507	5847774	5848778	Terpene_syn_2	Terpene	Unknown
	Region 1.2	Scaffold1	894368	913411		Terpene	Unknown
	Region 1.12	Scaffold1	6364297	6378951		Terpene	Unknown
	Region 2.4	Scaffold2	5325273	5339028		Terpene	Squalestatin S1 (40%)
	Region 3.2	Scaffold3	513702	535307		Terpene	Clavaric acid (100%)
	Region 3.3	Scaffold3	708836	728869		Terpene	Unknown
	Region 3.8	Scaffold3	3950752	3972575		Terpene	Unknown
	Region 4.1	Scaffold4.g35	162528	165233	Terpene_syn_2	Terpene	Botrydial (13%)
	Region 8.1	Scaffold8	21842	43461		Terpene	Unknown
	Region 3.11	Scaffold3.g1233	4587919	4591344	CaiC-P-Te	Other	Unknown
	Region 8.2	Scaffold8.g25	91098	92003	Te	Other	Unknown
	Region 8.3	Scaffold8.g317	1451503	1453313	CaiC	Other	Unknown
B. pseudobassiana	Region 1.2	Scaffold1.g284	1090776	1095288	C-A-C	NRPS	Unknown
	Region 1.3	Scaffold1.g285	1096607	1100811	C	NRPS	Unknown
	Region 1.4	Scaffold1.g1075	3916622	3920967	A-C	NRPS	Unknown
	Region 2.1	Scaffold2.g106	442121	451877	P-A	NRPS	Friulimicin A/B/C/D (9%)
	Region 2.2	Scaffold2.g107	453195	456640	C-P-C	NRPS	Unknown
	Region 2.6	Scaffold2.g279	1061584	1066363	A	NRPS	Unknown
	Region 2.12	Scaffold2.g944	3498712	3502754	A-C	NRPS	Unknown
	Region 3.1	Scaffold3.g154	602881	604833	A	NRPS	Unknown
	Region 3.4	Scaffold3.g764	2931393	2937187	A-C	NRPS	Unknown
	Region 3.7	Scaffold3.g1320	4924447	4928305	A-P-Te-Te	NRPS	Unknown
	Region 4.3	Scaffold4.g568	2094871	2098625	C-A-P-C	NRPS	Unknown

Region 5.4	Scaffold5.g493	1984194	1987843	C-A-P-C	NRPS	Myxochromide S (10%)
Region 5.5	Scaffold5.g494	1989011	1991272	A	NRPS	Myxochromide S (10%)
Region 5.6	Scaffold5.g518	2058622	2062464	A-P-T	NRPS	Unknown
Region 6.3	Scaffold6.g575	1999402	2012679	A-C-C-A-C	NRPS	Ferrichrome (66%)
Region 6.6	Scaffold6.g983	3535172	3538604	C-A-Te	NRPS	Unknown
Region 7.6	Scaffold7.g767	2723880	2729490	P-C-A-P	NRPS	Unknown
Region 7.7	Scaffold7.g768	2735215	2736921	P-C	NRPS	Unknown
Region 8.1	Scaffold8.g435	1565986	1571440	A-P-C-P-C	NRPS	Unknown
Region 11.1	Scaffold11.g41	155462	158417	A	NRPS	Unknown
Region 13.1	Scaffold13.g60	229571	230955	A	NRPS	Unknown
Region 13.2	Scaffold13.g62	233626	240776	C-A-P-C-A-Te	NRPS	Unknown
Region 30.1	Scaffold30.g2	4140	5222	A	NRPS	Unknown
Region 1.5	Scaffold1.g1078	3932022	3943816	KS-AT-DH-KR-P-C-A-Te	NRPS,PKS	Unknown
Region 2.11	Scaffold2.g815	3023261	3035707	KS-AT-DH-MT-KR-P-C-A-Te	NRPS,PKS	Unknown
Region 2.13	Scaffold2.g1160	4278566	4285005	A-P-KS-AT-KR-P	NRPS,PKS	Thuggacin A (15%)
Region 2.4	Scaffold2.g193	779731	781811	A-FNR	NRPS,Other	Unknown
Region 2.8	Scaffold2.g420	1582785	1587982	A-DhbB2-CYP	NRPS,Other	Unknown
Region 7.2	Scaffold7.g528	1893649	1899022	CaiC-C	NRPS,Other	Unknown
Region 3.6	Scaffold3.g1043	3965874	3973121	A-P-KS-AT-Fuz	PKS,Other	Unknown
Region 2.5	Scaffold2.g213	849139	857124	KS-AT-DH-MT-ER-KR-ACP	HR-PKS	Unknown
Region 2.7	Scaffold2.g383	1457354	1464405	KS-AT-DH-MT-ER-KR-ACP	HR-PKS	Unknown
Region 3.2	Scaffold3.g721	2748268	2755429	KS-AT-DH-ER-KR-ACP	HR-PKS	Unknown

	Region 4.1	Scaffold4.g6	16742	24583	KS-AT-DH-MT-ER-KR-ACP	HR-PKS	Unknown
	Region 4.2	Scaffold4.g162	585226	592559	KS-AT-DH-ER-KR	HR-PKS	Unknown
	Region 5.2	Scaffold5.g110	437359	444795	KS-AT-DH-ER-KR-ACP	HR-PKS	Unknown
	Region 6.5	Scaffold6.g820	2961911	2970201	KS-AT-DH-MT-ER-KR	HR-PKS	Unknown
	Region 7.5	Scaffold7.g764	2708864	2716614	KS-AT-DH-MT-ER-KR-ACP	HR-PKS	Unknown
	Region 3.3	Scaffold3.g761	2918634	2925994	KS-AT-DH-KR	PR-PKS	Unknown
	Region 1.8	Scaffold1.g1659	6056324	6059358	KS-AT	NR-PKS	Unknown
	Region 1.1	Scaffold1.g62	326961	327503	KS	NR-PKS	Unknown
	Region 2.9	Scaffold2.g563	2074744	2078718	KS-AT-PT	NR-PKS	Unknown
	Region 3.5	Scaffold3.g964	3679020	3679915	KS	NR-PKS	Unknown
	Region 5.3	Scaffold5.g467	1881589	1882023	KS	NR-PKS	Unknown
	Region 6.2	Scaffold6.g257	886074	890193	KS-AT-PT-ACP-Te	NR-PKS	Unknown
	Region 6.4	Scaffold6.g653	2293269	2295030	KS	NR-PKS	Unknown
	Region 7.1	Scaffold7.g3	16845	19460	AT-PT-ACP	NR-PKS	Unknown
	Region 7.4	Scaffold7.g608	2155272	2160819	SAT-KS-AT-ACP	NR-PKS	Unknown
	Region 1.6	Scaffold1	5324022	5343175		Terpene	Squalestatin S1 (40%)
	Region 1.7	Scaffold1	6013623	6034662		Terpene	Trichodiene-11-one (18%)
	Region 2.3	Scaffold2	658495	679091		Terpepe	Unknown
	Region 6.1	Scaffold6	139651	160253		Terpepe	Unknown
	Region 1.9	Scaffold1.g1660	6061023	6062109	Te	Other	Unknown
	Region 2.10	Scaffold2.g759	2807595	2812312	DIT1-CaiC-P-Transferase	Other	Unknown
	Region 5.1	Scaffold5.g64	271531	272343	Te	Other	Unknown
	Region 7.3	Scaffold7.g607	2154205	2154871	Te	Other	Unknown
B.	Region 1.1	AZHA01000001.g6	98445	99002	A-P	NRPS	Unknown

brongniartii	Region 1.3	AZHA01000001.g138	603998	618239	P-A	NRPS	Emericellamide A/B (60%)
	Region 3.3	AZHA01000003.g342	1228169	1234265	A-C	NRPS	Unknown
	Region 4.2	AZHA01000004.g13	59825	60001	A	NRPS	Unknown
	Region 6.1	AZHA01000006.g173	585669	587857	A-P-C	NRPS	Unknown
	Region 6.2	AZHA01000006.g174	587967	588931	A	NRPS	Unknown
	Region 6.4	AZHA01000006.g314	1054118	1059610	A-P-C-P-C	NRPS	Dimethylcoprogen (100%)
	Region 7.1	AZHA01000007.g297	956944	962661	C-A-P-C-P-C	NRPS	Unknown
	Region 11.1	AZHA01000011.g32	110495	117625	P-A-C	NRPS	Unknown
	Region 11.2	AZHA01000011.g52	185635	189666	A-P-T	NRPS	Unknown
	Region 11.3	AZHA01000011.g219	717898	724388	C-A-C-A-P-C-A	NRPS	Unknown
	Region 12.2	AZHA01000012.g84	292219	305733	A-A-P-C	NRPS	Unknown
	Region 15.1	AZHA01000015.g188	623773	632902	C-A-P-C-MT-P-C	NRPS	Bassianolide (60%)
	Region 18.2	AZHA01000018.g85	297933	302304	A-C	NRPS	Unknown
	Region 19.3	AZHA01000019.g141	463755	468010	A-C	NRPS	Unknown
	Region 22.1	AZHA01000022.g80	265954	269823	A-P-Te-Te	NRPS	Unknown
	Region 27.1	AZHA01000027.g37	120525	135222	P-A-C-P-C-P-C	NRPS	Unknown
	Region 29.1	AZHA01000029.g69	257185	265392	A-C-A-C-A-Te	NRPS	Unknown
	Region 31.1	AZHA01000031.g50	179897	182677	A-C-A-C-A-Te	NRPS	Unknown
	Region 40.2	AZHA01000040.g46	180547	183841	A	NRPS	Unknown
	Region 1.5	AZHA01000001.g360	1310057	1313713	CaiC-C	NRPS,Other	Unknown
	Region 2.3	AZHA01000002.g529	1706283	1709872	A-P-Lys2b	NRPS,Other	Unknown
	Region 5.1	AZHA01000005.g205	699800	705688	C-A-KS	NRPS,PKS	Unknown
	Region 18.1	AZHA01000018.g82	275216	287050	KS-AT-DH-MT-KR-P-C-A-P-Te	NRPS,PKS	Unknown
	Region 19.1	AZHA01000019.g11	33835	46589	KS-AT-DH-MT-KR-P-C-A-P-Te	NRPS,PKS	Fumosorinone (83%)

Region 1.4	AZHA01000001.g141	625469	633265	KS-AT-MT-ER-KR-ACP	HR-PKS	Emericellamide A/B (60%)
Region 2.2	AZHA01000002.g419	1305541	1312746	KS-AT-DH-MT-ER-KR-ACP	HR-PKS	Unknown
Region 2.4	AZHA01000002.g599	1920833	1928943	KS-AT-MT-ER-KR-ACP	HR-PKS	Unknown
Region 3.1	AZHA01000003.g296	1044474	1052072	KS-AT-DH-ER-KR-ACP	HR-PKS	Unknown
Region 4.1	AZHA01000004.g8	31792	39465	KS-AT-DH-ER-KR-ACP	HR-PKS	Unknown
Region 6.3	AZHA01000006.g196	648517	656875	KS-AT-DH-MT-ER-KR-ACP	HR-PKS	Unknown
Region 12.1	AZHA01000012.g5	17217	24863	KS-AT-DH-ER-KR-ACP	HR-PKS	Unknown
Region 19.2	AZHA01000019.g74	224793	232361	KS-AT-DH-ER-KR-ACP	HR-PKS	Unknown
Region 20.1	AZHA01000020.g16	53098	61703	KS-AT-MT-ER-KR-ACP	HR-PKS	Unknown
Region 25.2	AZHA01000025.g121	413511	421080	KS-AT-DH-ER-KR	HR-PKS	4-epi-15-epi-brefeldin A (50%)
Region 3.2	AZHA01000003.g339	1215198	1222434	KS-AT-KR-ACP	PR-PKS	Unknown
Region 25.1	AZHA01000025.g98	336197	341608	KS-AT-DH-KR-ACP	PR-PKS	6-methylsalicyclic acid (100%)
Region 1.2	AZHA01000001.g75	392188	399370	SAT-KS-AT-PT-ACP-ACP-Te	NR-PKS	Unknown
Region 2.1	AZHA01000002.g222	706086	711397	KS-AT-PT-ACP-ACP-Te	NR-PKS	bikaverin (57%)
Region 5.2	AZHA01000005.g287	989442	990785	KS	NR-PKS	Unknown
Region 26.1	AZHA01000026.g80	287098	293860	SAT-KS-AT-PT-ACP-ACP-Te	NR-PKS	Neosartorin (15%)
Region 35.2	AZHA01000035.g73	222132	223531	AT	NR-PKS	Unknown
Region 24.1	AZHA01000024	37469	58914		Terpene	Unknown
Region 35.1	AZHA01000035	66175	88181		Terpene	Unknown
Region 40.3	AZHA01000040	56475	78250		Terpene	Unknown
Region 41.1	AZHA01000041.g37	146936	149696	Terpene_syn_C_2	Terpene	Mangicol A (33%)
Region 40.1	AZHA01000040	6114	16767		siderophore	Unknown

	Region 6.5	AZHA01000006.g315	1062070	1063874	CaiC	Other	Unknown
	Region 24.2	AZHA01000024.g33	106124	107023	Te	Other	Unknown
	Region 42.1	AZHA01000042.g35	94784	99670	DITI-CaiC-P-Transferase	Other	Unknown
L. fungicola	Region 21.2	FWCC01000021.g14	55518	58615	A-Te	NRPS	Phomasetin (42%)
	Region 40.1	FWCC01000040.g18	51339	56337	P-C-A-C	NRPS	Unknown
	Region 43.1	FWCC01000043.g53	174803	178661	A-P-T	NRPS	Unknown
	Region 52.1	FWCC01000052.g43	150573	165042	A-A-P-P-C	NRPS	Unknown
	Region 66.1	FWCC01000066.g25	89853	92693	A-P-Te	NRPS	Unknown
	Region 73.1	FWCC01000073.g5	13489	18357	A-P-Te-Te	NRPS	Unknown
	Region 79.1	FWCC01000079.g16	43205	47544	A-C	NRPS	Unknown
	Region 82.1	FWCC01000082.g107	396991	402311	A-C	NRPS	Unknown
	Region 91.1	FWCC01000091.g21	42594	55840	C-A-A-P	NRPS	Unknown
	Region 116.1	FWCC01000116.g21	60631	64680	A-P-T	NRPS	Unknown
	Region 126.1	FWCC01000126.g3	10173	16034	A-P-C-P-C	NRPS	Dimethylcoprogen (100%)
	Region 164.2	FWCC01000164.g42	142511	182549	A-A-P-C-A-P-A-P-A-P-A-C	NRPS	Unknown
	Region 174.1	FWCC01000174.g8	27048	47966	A-P-C-P-A-A-C	NRPS	Unknown
	Region 220.1	FWCC01000220.g24	69171	72038	A-P-Te	NRPS	Unknown
	Region 232.2	FWCC01000232	132493	175813		NRPS	Unknown
	Region 241.1	FWCC01000241.g66	232720	237817	C-A-P-C	NRPS	Acetylaranotin (60%)
	Region 247.1	FWCC01000247.g17	67302	72205	A-C-A	NRPS	Unknown
	Region 247.2	FWCC01000247.g18	75474	86807	A-C	NRPS	Unknown
	Region 278.2	FWCC01000278.g20	74469	78784	A-C	NRPS	Unknown
	Region 278.3	FWCC01000278.g21	82598	86035	A-P-C	NRPS	Unknown
	Region 296.1	FWCC01000296	13348	57472		NRPS	Unknown
	Region 345.1	FWCC01000345.g22	81538	84936	C-A	NRPS	Unknown

Region 377.1	FWCC01000377.g31	88337	103000	A-C-A-Te	NRPS	Beauvericin (30%)
Region 409.1	FWCC01000409.g14	36406	40752	A-C	NRPS	Unknown
Region 507.1	FWCC01000507.g4	5415	9731	A-C	NRPS	Unknown
Region 37.1	FWCC01000037	1	6735		NRPS-like	Unknown
Region 252.1	FWCC01000252	36861	70688		NRPS-like	Unknown
Region 79.2	FWCC01000079.g19	61190	73027	KS-AT-DH-MT-KR-P-C-A-P-Te	NRPS,PKS	Unknown
Region 121.1	FWCC01000121.g39	105148	113678	KS-AT-DH-KR-P-C	NRPS,PKS	Unknown
Region 278.1	FWCC01000278.g4	15286	27644	KS-AT-DH-MT-KR-P-C-A-Te	NRPS,PKS	Equisetin (27%)
Region 350.1	FWCC01000350.g15	48203	56685	C-A-KS	NRPS,PKS	Unknown
Region 375.2	FWCC01000375.g23	73337	85499	KS-AT-MT-KR-P-C-A-P-Te	NRPS,PKS	Fusarielin H (25%)
Region 114.1	FWCC01000114.g50	151692	164184	A-P-A-Te-CzcO	NRPS,Other	Unknown
Region 177.1	FWCC01000177.g31	95847	117100	ANK-MT-A-P-C-P-P-A	NRPS,Other	Unknown
Region 304.1	FWCC01000304.g11	31735	35507	A-Lys2b	NRPS,Other	Unknown
Region 304.2	FWCC01000304.g24	71648	74929	A-NOX	NRPS,Other	Unknown
Region 307.1	FWCC01000307.g3	4315	8154	CaiC-C	NRPS,Other	Unknown
Region 64.1	FWCC01000064.g30	122001	126169	AP3_Mu_N-Adap_comp_sub-AT	PKS,Other	Unknown
Region 85.1	FWCC01000085.g53	171129	179972	KS-AT-DH-MT-ER-KR-ACP-Carn_acyltransf	PKS,Other	Sordarin (8%)
Region 177.2	FWCC01000177.g81	278045	280997	KS-Ubiq_cyt_C_chap	PKS,Other	Unknown
Region 283.1	FWCC01000283.g12	36234	45877	KS-AT-MT-ER-KR-ACP-Lactamase	PKS,Other	Unknown

Region 51.1	FWCC01000051.g12	33022	40245	KS-AT-DH-ER-KR-ACP-Te	HR-PKS	Unknown
Region 91.2	FWCC01000091.g22	58518	68944	KS-AT-DH-MT-ER-KR-ACP-CYP	HR-PKS	Unknown
Region 159.1	FWCC01000159.g1	90	6550	AT-DH-MT-ER-KR-ACP	HR-PKS	Unknown
Region 296.2	FWCC01000296.g31	114775	122112	KS-AT-DH-ER-KR-ACP	HR-PKS	Unknown
Region 333.1	FWCC01000333.g12	37105	45286	KS-AT-DH-MT-ER-KR-ACP	HR-PKS	Brasilinolide A/B/C (2%)
Region 343.1	FWCC01000343.g19	79787	88063	KS-AT-DH-MT-ER-KR-ACP	HR-PKS	Asperfuranone (45%)
Region 372.1	FWCC01000372.g19	49717	57409	KS-AT-DH-MT-ER-KR-ACP	HR-PKS	Unknown
Region 375.1	FWCC01000375.g7	26070	34102	AT-MT-ER-KR-ACP	HR-PKS	E-837 (50%)
Region 399.1	FWCC01000399.g23	72670	79995	KS-AT-DH-ER-KR-ACP	HR-PKS	4-epi-15-epi-brefeldin A (50%)
Region 402.1	FWCC01000402.g1	4430	11519	KS-AT-DH-MT-ER-KR	HR-PKS	Unknown
Region 408.1	FWCC01000408.g4	6677	15703	Te-KS-AT-ER-KR	HR-PKS	Pyranonigrin E (100%)
Region 595.1	FWCC01000595.g4	15076	24024	KS-AT-DH-MT-ER-KR-ACP	HR-PKS	Unknown
Region 25.1	FWCC01000025.g20	69151	77286	KS-AT-DH-MT-KR-ACP	PR-PKS	Unknown
Region 164.1	FWCC01000164.g37	116273	123395	KS-AT-DH-ER-Te	PR-PKS	Unknown
Region 283.2	FWCC01000283.g37	112659	120574	KS-AT-DH-MT-KR-ACP-Te	PR-PKS	Fujikurin A/B/C/D (50%)
Region 21.1	FWCC01000021.g12	46690	51144	KS-AT-DH-MT	NR-PKS	Phomasetin (42%)
Region 94.1	FWCC01000094.g10	44506	45998	KS	NR-PKS	Unknown
Region 156.1	FWCC01000156.g3	8916	14884	KS-AT-PT-ACP-HTH-MT	NR-PKS	Citrinin (43%)
Region 158.1	FWCC01000158.g19	52015	56255	KS-AT-PT-ACP	NR-PKS	Neosartorin (42%)

	Region 287.1	FWCC01000287.g1	370	1101	KS	NR-PKS	Unknown
	Region 305.1	FWCC01000305.g29	98669	106364	SAT-KS-PT-ACP-HTH-MT-Aes-MhpC	NR-PKS	Unknown
	Region 343.2	FWCC01000343.g24	99843	108485	KS-AT-PT-ACP-ACP-HTH-MT-Te	NR-PKS	Asperfuranone (45%)
	Region 341.1	FWCC01000341.g2	3116	6688	CYP-SAT-KS	NR-PKS	Unknown
	Region 341.2	FWCC01000341.g21	85110	91209	KS-AT-PT-ACP-ACP-Te	NR-PKS	Naphthopyrone (100%)
	Region 381.1	FWCC01000381.g1	337	1580	KS	NR-PKS	Unknown
	Region 382.1	FWCC01000382.g1	337	1624	KS	NR-PKS	Unknown
	Region 461.1	FWCC01000461.g6	22825	23293	KS	NR-PKS	Unknown
	Region 485.1	FWCC01000485.g9	21924	26188	AT-PT-ACP-Te	NR-PKS	Unknown
	Region 527.1	FWCC01000527.g2	3018	8649	KS-AT-PT-ACP-ACP-Te	NR-PKS	Unknown
	Region 604.1	FWCC01000604.g9	19050	21393	KS-AT	NR-PKS	Unknown
	Region 571.1	FWCC01000571	1	10831		PKS	Naphthopyrone (100%)
	Region 117.1	FWCC01000117	94444	115511		Terpene	Squalestatin S1 (40%)
	Region 232.1	FWCC01000232.g2	2638	4460	Terpene_syn_C_2	Terpene	Unknown
	Region 51.2	FWCC01000051.g33	109045	109977	Te	Other	Unknown
	Region 81.1	FWCC01000081.g7	19984	23338	CaiC-Te	Other	Unknown
L. psalliotae	Region 8.2	PHFE01000008.g472	1536386	1540633	A-C	NRPS	Unknown
	Region 8.4	PHFE01000008.g1309	4275053	4289544	P-A-C-P-C-P-C	NRPS	Unknown
	Region 28.2	PHFE01000028.g65	242164	246587	A-C	NRPS	Unknown
	Region 28.4	PHFE01000028.g481	1655133	1658999	A-P-Te-Te	NRPS	Unknown
	Region 29.1	PHFE01000029.g45	143325	148520	A-P-C-A-Te	NRPS	Unknown
	Region 29.2	PHFE01000029.g416	1335759	1339808	A-P-T	NRPS	Unknown
	Region 29.5	PHFE01000029.g591	1862195	1872169	A-P-A-Te	NRPS	Unknown
	Region 45.1	PHFE01000045.g11	33992	37213	A-P-Te	NRPS	Unknown

Region 46.1	PHFE01000046.g29	88009	91261	A	NRPS	Unknown
Region 51.1	PHFE01000051.g1	5033	7234	A	NRPS	Unknown
Region 55.1	PHFE01000055.g36	106127	120925	A-A-P-Te	NRPS	Unknown
Region 66.1	PHFE01000066.g3	10671	15380	A-C	NRPS	Unknown
Region 81.1	PHFE01000081.g100	303106	308506	P-C-A	NRPS	Unknown
Region 92.1	PHFE01000092.g26	94225	107316	A-P-A-C-A-P-C-P	NRPS	Leucinostatin A/B (10%)
Region 93.2	PHFE01000093	1550261	1593626		NRPS	Unknown
Region 95.2	PHFE01000095.g336	1071627	1077074	A-P-C-P-C	NRPS	Dimethylcoprogen (100%)
Region 107.1	PHFE01000107.g327	1011389	1032325	A-P-A-P-A	NRPS	Unknown
Region 91.1	PHFE01000091	256483	298630		NRPS-like	Unknown
Region 28.1	PHFE01000028.g68	258340	270180	KS-AT-DH-MT-KR-P-C-A-P-Te	NRPS,PKS	Unknown
Region 31.1	PHFE01000031.g35	148002	160300	KS-AT-DH-KR-P-C-A-Te	NRPS,PKS	Dihydroisoflavipucine/isoflavipucine (25%)
Region 107.3	PHFE01000107.g672	2108474	2120331	KS-AT-DH-ER-KR-P-C-A-P-Te	NRPS,PKS	Fusarin (100%)
Region 110.1	PHFE01000110.g318	982641	982687	A-P-Lys2b	NRPS,Other	Unknown
Region 107.4	PHFE01000107.g817	2587519	2598434	KS-AT-DH-MT-ER-KR-SAS	PKS,Other	Squalestatin S1 (40%)
Region 8.3	PHFE01000008.g907	2965673	2972896	KS-AT-DH-ER-KR-ACP	HR-PKS	Unknown
Region 28.3	PHFE01000028.g426	1490319	1497208	KS-AT-DH-ER-KR-ACP	HR-PKS	Unknown
Region 51.3	PHFE01000051.g478	1460760	1468036	KS-AT-ER-KR-ACP	HR-PKS	Alternapyrone (40%)
Region 83.1	PHFE01000083.g158	445181	454941	KS-AT-MT-ER-KR	HR-PKS	Unknown
Region 86.1	PHFE01000086.g29	94937	102574	KS-AT-DH-ER-KR-ACP	HR-PKS	Unknown
Region 93.3	PHFE01000093.g785	2483923	2491744	KS-AT-DH-ER-KR-ACP	HR-PKS	Fumonisin (11%)
Region 8.1	PHFE01000008.g273	913266	920867	KS-AT-KR-ACP	PR-PKS	FujikurinA/B/C/D (50%)

	Region 55.2	PHFE01000055.g944	2899192	2908214	Te-KS-AT-DH-ER-ACP	PR-PKS	Pyranonigrin E (100%)
	Region 66.2	PHFE01000066.g6	20095	29245	ER-KS-AT-DH-KR-ACP	PR-PKS	Unknown
	Region 51.2	PHFE01000051.g235	716459	723030	SAT-KS-AT-PT-ACP-Te	NR-PKS	Unknown
	Region 75.1	PHFE01000075.g24	89996	96665	SAT-KS-AT-PT-ACP-ACP-Te	NR-PKS	1,3,6,8-tetrahydroxynaphthalene (100%)
	Region 91.2	PHFE01000091.g301	914613	919554	KS-PT-ACP	NR-PKS	Unknown
	Region 93.1	PHFE01000093.g416	1355250	1362377	SAT-KS-AT-PT-ACP-ACP-Te	NR-PKS	Unknown
	Region 110.2	PHFE01000110.g336	1046530	1056165	SAT-KS-AT-ACP-Te	NR-PKS	Unknown
	Region 22.1	PHFE01000022	61300	79269		Terpene	Unknown
	Region 22.2	PHFE01000022	544786	566669		Terpene	Nivalenol / deoxynivalenol / 3-acetyldeoxynivalenol / 15-acetyldeoxynivalenol / neosolaniol calonectrin / apotichodiol /isotrichotriol l15-decalonectrin / T-2 toxin / 3-acetyl T-2 toxin / trichodiene (8%)
	Region 29.3	PHFE01000029	1668908	1690275		Terpene	Unknown
	Region 95.1	PHFE01000095	784171	804482		Terpene	Unknown
	Region 29.4	PHFE01000029.g555	1752749	1753645	Te	Other	Unknown
	Region 98.1	PHFE01000098.g35	97395	102268	DIT1-CaiC-P-PLN02481	Other	Unknown
	Region 107.2	PHFE01000107.g665	2095542	2096474	Te	Other	Unknown
Simplicillium aogashimaense	Region 2.1	JAALXG010000002.g259	780824	782749	CaiC-C	NRPS,Other	Unknown
	Region 2.4	JAALXG010000002	3461681	3509591		NRPS	Unknown
	Region 2.5	JAALXG010000002.g1403	4591947	4599416	A-P-C-A-P-Te	NRPS	Ergotamine (23%)
	Region 3.1	JAALXG010000003.g38	152140	158402	A-P-C-P-C	NRPS	Unknown
	Region 4.3	JAALXG010000004.g224	722527	725587	C-A-MT	NRPS	Unknown

Region 4.4	JAALXG010000004.g225	731791	734739	A-MT	NRPS	Unknown
Region 4.5	JAALXG010000004.g388	1242009	1273722	C-A-P-C-P-P-C-P-C-P-C-P-C	NRPS	KK-1 (20%)
Region 5.2	JAALXG010000005.g350	1253916	1280164	P-C-A-A-C-C-A-C	NRPS	Unknown
Region 5.4	JAALXG010000005.g519	1953835	1958103	A-P-C-P-C	NRPS	Unknown
Region 6.4	JAALXG010000006.g412	1347032	1356856	A-C-A-C-P-C-P-C	NRPS	Unknown
Region 7.1	JAALXG010000007.g178	586429	597492	A-P-A-P-C-Te	NRPS	Cephalosporin C (71%)
Region 8.1	JAALXG010000008.g6	41662	44499	A-P-Te	NRPS	Stipitatic acid (14%)
Region 8.6	JAALXG010000008.g337	1047818	1060180	A-P-C	NRPS	Unknown
Region 11.2	JAALXG010000011.g100	333113	336958	A-P-Te-Te	NRPS	Unknown
Region 11.3	JAALXG010000011.g604	2025735	2036907	P-A-P	NRPS	Bassianolide (13%)
Region 14.1	JAALXG010000014.g81	270319	297084	A-P-P-A-A-A-P-C	NRPS	Unknown
Region 14.2	JAALXG010000014.g84	308150	343966	P-A-P-A-A-P-A-A-A-C	NRPS	Unknown
Region 2.2	JAALXG010000002	871371	914118		NRPS-like	Unknown
Region 8.7	JAALXG010000008	1671367	1714498		NRPS-like	Unknown
Region 1.1	JAALXG010000001.g10	79188	91416	KS-AT-DH-MT-KR-P-C-A-Te	NRPS,PKS	Equisetin (18%)
Region 5.3	JAALXG010000005.g403	1458017	1461774	A-P-Lys2b	NRPS,Other	Unknown
Region 8.5	JAALXG010000008.g177	561056	566207	CaiC-P-T	NRPS,Other	Unknown
Region 11.1	JAALXG010000011.g32	111242	113771	A-NOX	NRPS,Other	Unknown
Region 7.2	JAALXG010000007.g190	647448	654956	KS-AT-ER-KR	HR-PKS	Unknown
Region 8.2	JAALXG010000008.g20	89350	97007	KS-AT-DH-MT-ER-KR-ACP	HR-PKS	Unknown
Region 10.1	JAALXG010000010.g54	175512	183607	KS-AT-DH-ER-KR-ACP	HR-PKS	Unknown
Region 11.4	JAALXG010000011.g653	2201858	2210289	KS-AT-DH-MT-ER-KR-ACP	HR-PKS	Unknown

Region 5.1	JAALXG010000005.g344	1231969	1236840	KS-AT-MT-KR	PR-PKS	Bll-rafflesfungin (15%)
Region 6.1	JAALXG010000006.g152	507711	516046	KS-AT-DH-MT-ER-ACP	PR-PKS	Unknown
Region 6.5	JAALXG010000006.g435	1444041	1449614	KS-AT-KR-ACP	PR-PKS	6-methylsalicyclic acid (100%)
Region 9.1	JAALXG010000009.g165	576327	581984	AT-DH-ER-ACP	PR-PKS	Unknown
Region 1.2	JAALXG010000001.g61	233591	234543	AT	NR-PKS	Unknown
Region 4.1	JAALXG010000004.g16	58471	65608	KS-AT-PT-ACP-ACP-HTH-MT-Aes	NR-PKS	Unknown
Region 6.3	JAALXG010000006.g354	1151790	1159830	KS-AT-PT-ACP-ACP-HTH-MT-Aes	NR-PKS	Cichorine (100%)
Region 6.6	JAALXG010000006.g584	1939103	1944621	KS-AT-PT-ACP-Te	NR-PKS	Naphthopyrone (100%)
Region 7.3	JAALXG010000007.g507	1748052	1753749	KS-AT-PT-ACP-ACP-Te	NR-PKS	6-hydroxymellein (33%)
Region 9.2	JAALXG010000009.g166	582814	583888	KS	NR-PKS	Unknown
Region 11.6	JAALXG010000011.g945	3221972	3228519	SAT-KS-AT-PT-ACP-ACP-Te	NR-PKS	Duclauxin (28%)
Region 14.3	JAALXG010000014.g301	1047068	1052980	SAT-KS-AT-PT-ACP-ACP-Te	NR-PKS	Viriditoxin (33%)
Region 6.2	JAALXG010000006	902868	948495		PKS	Unknown
Region 3.2	JAALXG010000003	688812	709984		Terpene	Unknown
Region 4.2	JAALXG010000004	490248	511369		Terpene	Unknown
Region 5.5	JAALXG010000005	3118204	3161382		Terpene	Nivalenol/deoxynivalenol/3-acetyldeoxynivalenol/15-acetyldeoxynivalenol/neosolaniol/calonectrin/apotrichodiol/isotrichotriol/15-decalonectrin/T-2 toxin/3-acetyl T-2 toxin/trichodiene (12%)
Region 8.8	JAALXG010000008	3062370	3083704		Terpene	Squalestatin S1 (40%)

	Region 11.5	JAALXG010000011	2317543	2339300		Terpene	Unknown
	Region 2.3	JAALXG010000002	978795	999904		indole	Unknown
	Region 8.4	JAALXG010000008	409770	432502		betalactone	Unknown
	Region 10.2	JAALXG010000010	1281325	1312244		NAPAA	Unknown
	Region 8.3	JAALXG010000008.g105	339053	340924	P-Te	Other	Unknown
G. kalimantanensis	Region 4.2	JAKLMQ010000004.g146	483109	491263	A-C	NRPS	Pladienolide B (37%)
	Region 12.1	JAKLMQ010000012.g18	51894	56150	A-C	NRPS	Unknown
	Region 23.1	JAKLMQ010000023.g104	349749	353639	A-P-T	NRPS	Unknown
	Region 25.1	JAKLMQ010000025.g59	196596	207662	A-P-A-P-Te	NRPS	Cephalosporin C (71%)
	Region 25.2	JAKLMQ010000025.g71	247415	251263	A-P-Te-Te	NRPS	Cephalosporin C (71%)
	Region 30.1	JAKLMQ010000030.g38	135241	147114	A-A-C-C-P-C	NRPS	Unknown
	Region 43.1	JAKLMQ010000043.g14	43376	46798	A	NRPS	Unknown
	Region 52.1	JAKLMQ010000052.g57	202045	210324	A-P-C-P-C	NRPS	Unknown
	Region 59.1	JAKLMQ010000059.g51	175149	176768	A	NRPS	Unknown
	Region 59.2	JAKLMQ010000059.g52	182218	185064	A-MT	NRPS	Unknown
	Region 62.1	JAKLMQ010000062.g38	107735	110702	A-P-Te	NRPS	Unknown
	Region 70.1	JAKLMQ010000070.g13	47953	52149	A-Te	NRPS	Unknown
	Region 77.2	JAKLMQ010000077.g24	86782	92331	A-P-C-P-C	NRPS	Dimethylcoprogen (100%)
	Region 4.1	JAKLMQ010000004.g52	173491	181101	KS-AT-ER-KR-ACP	HR-PKS	Pladienolide B (37%)
	Region 7.1	JAKLMQ010000007.g154	534178	550375	KS-AT-DH-ER-KR-ACP	HR-PKS	Communesin A/B/C/D/E/G/H (12%)
	Region 20.1	JAKLMQ010000020.g93	327548	335807	KS-AT-DH-MT-ER-KR-ACP	HR-PKS	Cyclosporin C (15%)
	Region 30.2	JAKLMQ010000030.g92	322855	330842	KS-AT-DH-MT-ER-KR-ACP	HR-PKS	Leucinostatin A/B (10%)
	Region 62.2	JAKLMQ010000062.g49	145033	151956	KS-AT-DH-ER-KR	HR-PKS	Phenalamide (50%)
	Region 15.1	JAKLMQ010000015.g104	330690	338569	KS-AT-DH-MT-KR-ACP	PR-PKS	Unknown

Region 6.1	JAKLMQ010000006.g196	662138	664542	KS	NR-PKS	Unknown
Region 21.1	JAKLMQ010000021.g71	210413	214118	AT-PT-ACP-HTH-MT	NR-PKS	Unknown
Region 26.1	JAKLMQ010000026.g125	413545	414941	KS	NR-PKS	Unknown
Region 34.2	JAKLMQ010000034.g35	140523	141066	KS	NR-PKS	Unknown
Region 1.1	JAKLMQ010000001.g266	947122	950894	A-Lys2b	NRPS,Other	Unknown
Region 3.1	JAKLMQ010000003.g286	901067	903756	CaiC-C	NRPS,Other	Unknown
Region 2.4	JAKLMQ010000002.g293	937134	949006	KS-AT-DH-ER-KR-P-C-A-P-Te	NRPS,PKS	Fusarin (100%)
Region 12.2	JAKLMQ010000012.g71	229603	241762	KS-AT-DH-MT-KR-P-C-A-Te	NRPS,PKS	Equisetin (36%)
Region 22.1	JAKLMQ010000022.g128	402429	408248	MT-KR-P-C	NRPS,PKS	Unknown
Region 34.1	JAKLMQ010000034.g27	91035	103522	KS-AT-DH-MT-KR-P-C-A-Te	NRPS,PKS	Cytochalasin E (30%)
Region 37.1	JAKLMQ010000037.g22	59653	71885	KS-AT-DH-MT-KR-P-C-A-Te	NRPS,PKS	Curvupallide-B (55%)
Region 58.1	JAKLMQ010000058.g33	101913	114215	KS-AT-DH-MT-KR-P-C-A-P-Te	NRPS,PKS	Equisetin (18%)
Region 2.2	JAKLMQ010000002.g271	870923	874488	AP3-Adap-AT	PKS,Other	Unknown
Region 2.1	JAKLMQ010000002.g264	845617	855559	A-P-KS-AT-KR-P-Te-Glyco	NRPS,PKS,Other	Codaside A/B (14%)
Region 11.1	JAKLMQ010000011.g75	256062	257030	Terpene_syn_C_2	Terpene	Unknown
Region 13.1	JAKLMQ010000013	14788	35768		Terpene	Squalestatin S1 (40%)
Region 2.3	JAKLMQ010000002.g288	927328	928254	Te	Other	Unknown
Region 8.1	JAKLMQ010000008.g66	219386	221423	CaiC	Other	Unknown
Region 12.3	JAKLMQ010000012.g135	467118	468229	Te	Other	Sorangicin A (26%)
Region 77.1	JAKLMQ010000077.g23	83149	84725	CaiC	Other	Unknown

Table S6. Overview of biosynthetic gene clusters in the genomes of the fourteen studied fungi.

**Species (NCBI)**	**No of cluster**	**NR<br>PS**	**NPRS-<br>like**	**PKS<br>(Total)**	**HR-<br>PKS**	**PR-<br>PKS**	**NR-<br>PKS**	**T3P<br>KS**	**Terp<br>ene**	**Hybrid<br>PKS-NRPS**	**Hybrid<br>NRPS-<br>Other**	**Hybrid PKS-<br>NRPS-Other**	**Hybri<br>d<br>PKS-<br>Other**	**Othe<br>r**
S. hepiali ICMM 82-2	54	19	1	16	6	3	7	1	3	9	2		1	2
S. hepiali FENG	57	22	1	12	7	2	3	1	3	12	1		1	4
S. yunnanensis YFCC 1527	58	21	1	14	7	3	5	1	4	8	5		1	2
A. lecanii	46	21	2	9	5		3		4	5	4		1	
C. cicadae	31	15		5	2	1	2		3	5	2			1
C. javanica	51	19	2	14	9	2	4		2	6	4	1	1	1
C. fumosorosea	54	25	3	9	3	1	5		3	7	3			4
B. bassiana	54	19		14	8	2	4	1	9	4	4			3
B. pseudobassiana	56	23		18	8	1	9		4	3	3		1	4
B. brongniartii	50	20		17	10	2	5		4	3	2			4
L. fungicola	76	25	2	31	12	3	15		2	5	5		4	2
L. psalliotae	44	17	1	14	6	3	5		4	3	1		1	3

Simplicillium aogashimaense	50	16	2	17	4	4	8	5	1	4			4
G. kalimantanensis	39	13		9	5	1	4	2	6	2	1	1	4

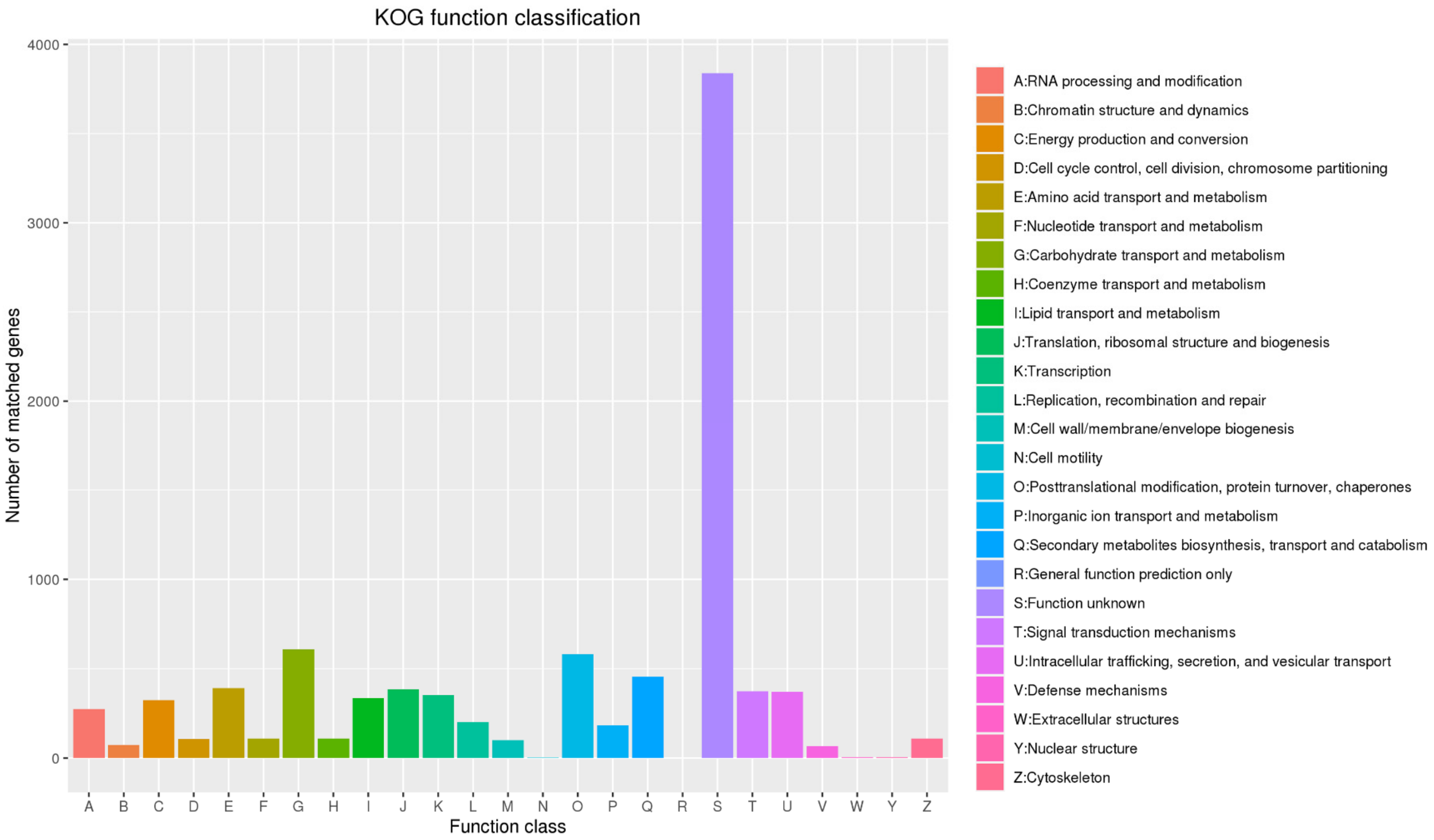

Figure S1. The biological function of functional genes involved in the annotation of *S. yunnanensis* YFCC 1527 in the EggNOg analysis.

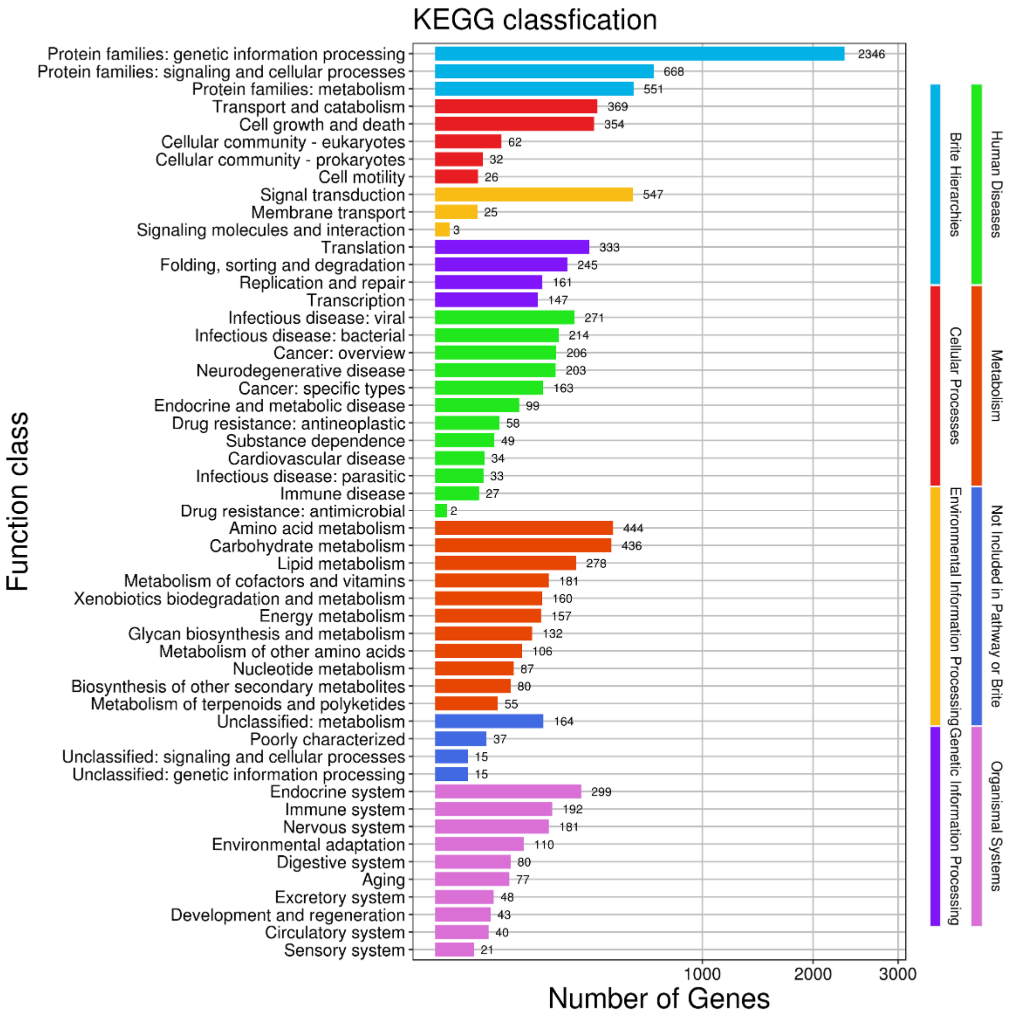

Figure S2. The biological function of functional genes involved in the annotation of *S. hepiali* ICMM 82-2 in the KEGG analysis.

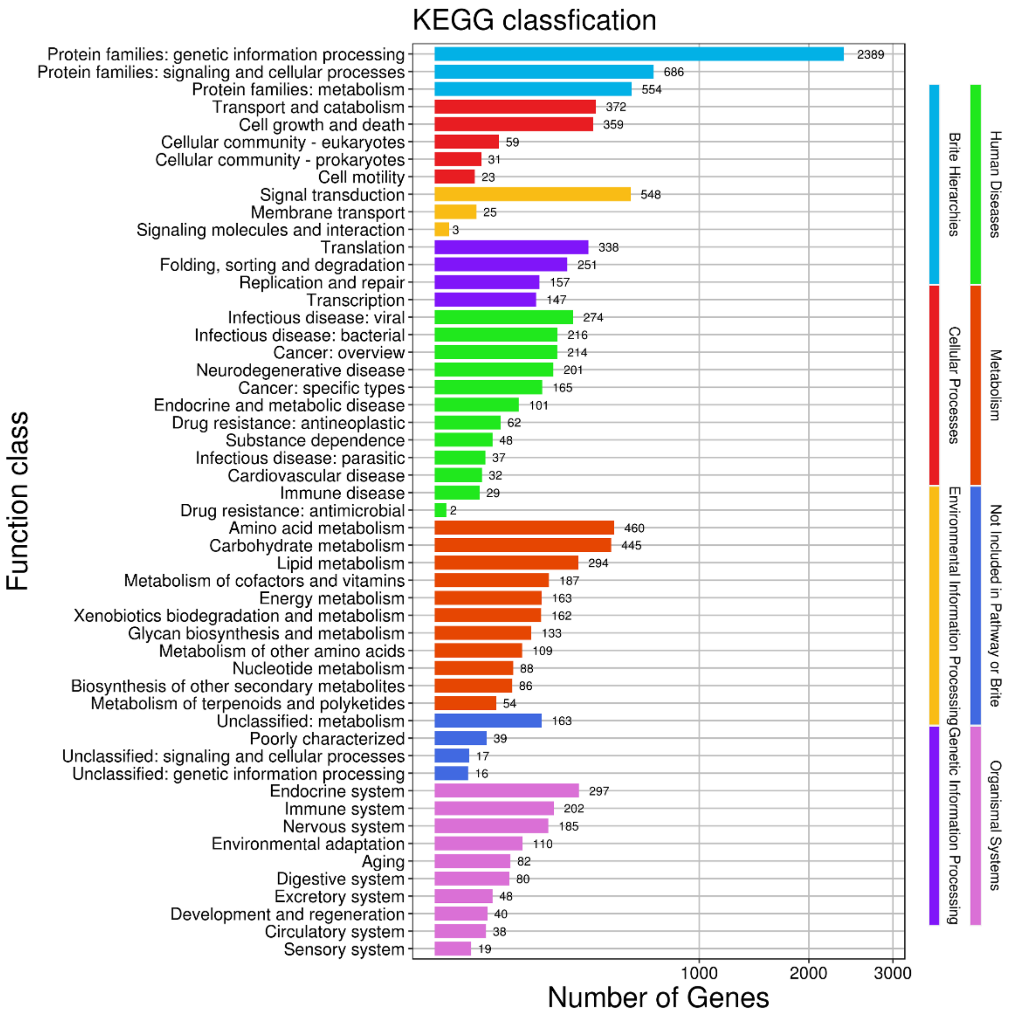

Figure S3. The biological function of functional genes involved in the annotation of *S. yunnanensis* YFCC 1527 in the KEGG analysis.

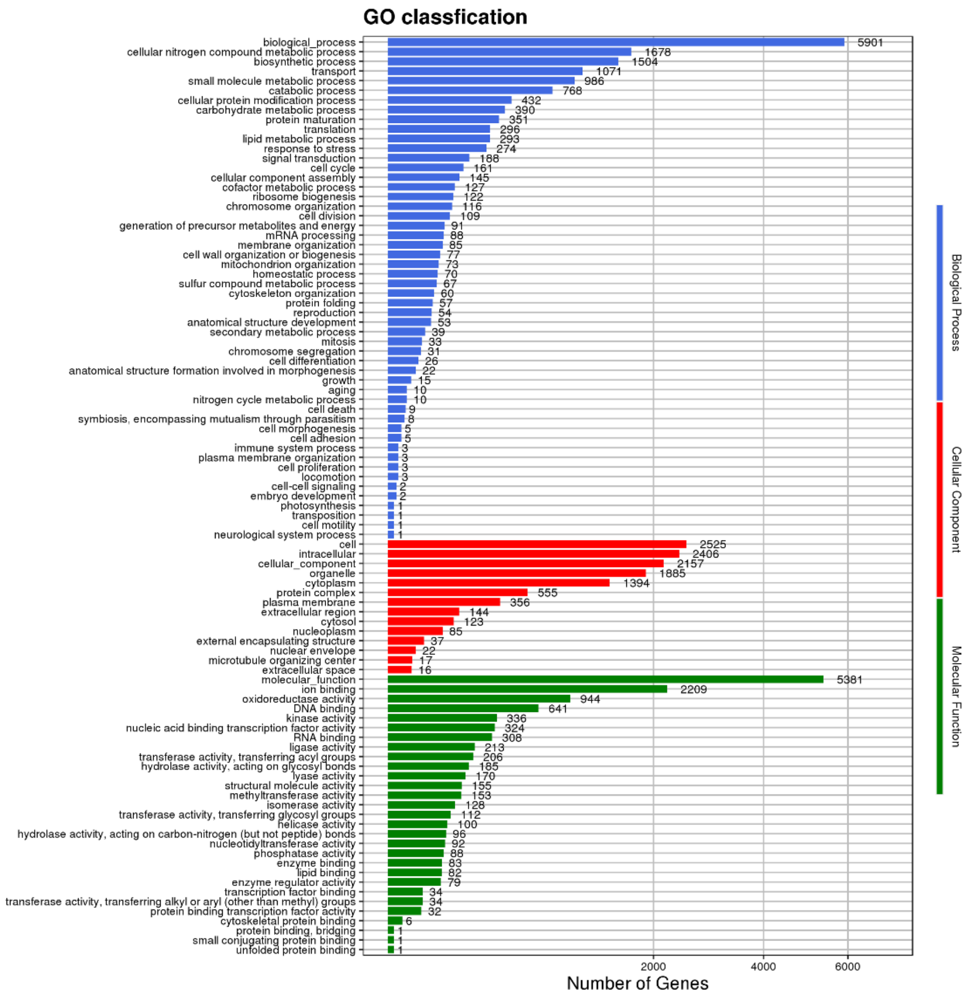

Figure S4. The biological function of functional genes involved in the annotation of *S. hepiali* ICMM 82-2 in the GO analysis.

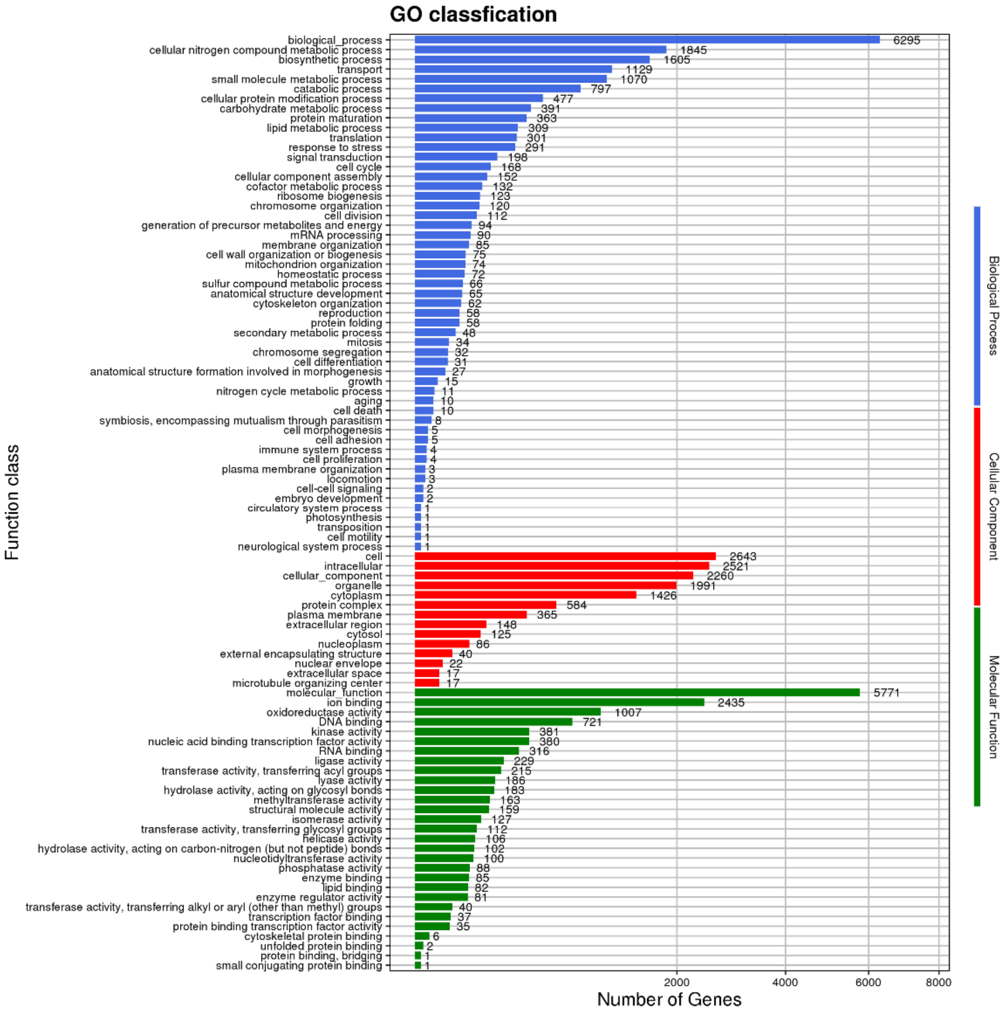

Figure S5. The biological function of functional genes involved in the annotation of *S. yunnanensis* YFCC 1527 in the GO analysis.

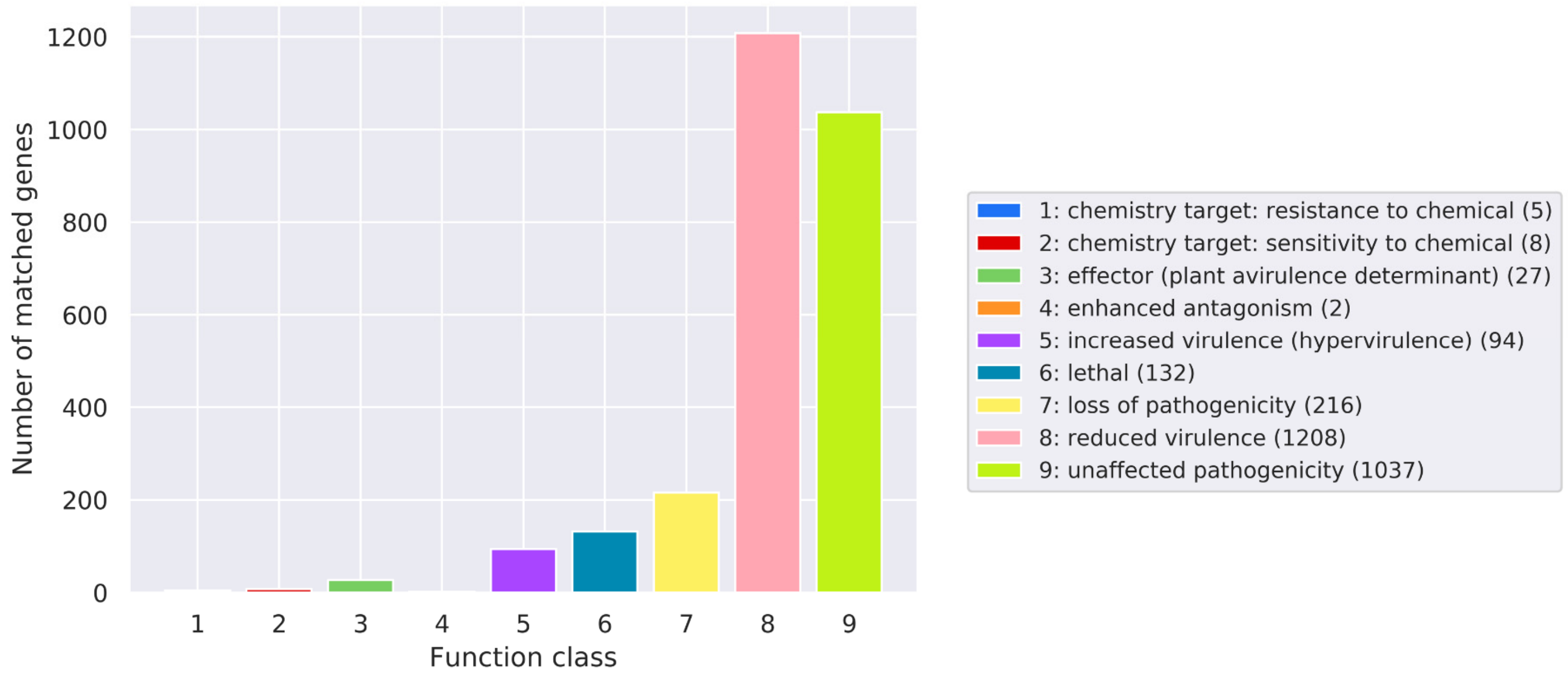

Figure S6. The biological function of functional genes involved in the annotation of *S. hepiali* ICMM 82-2 in the PHI analysis.

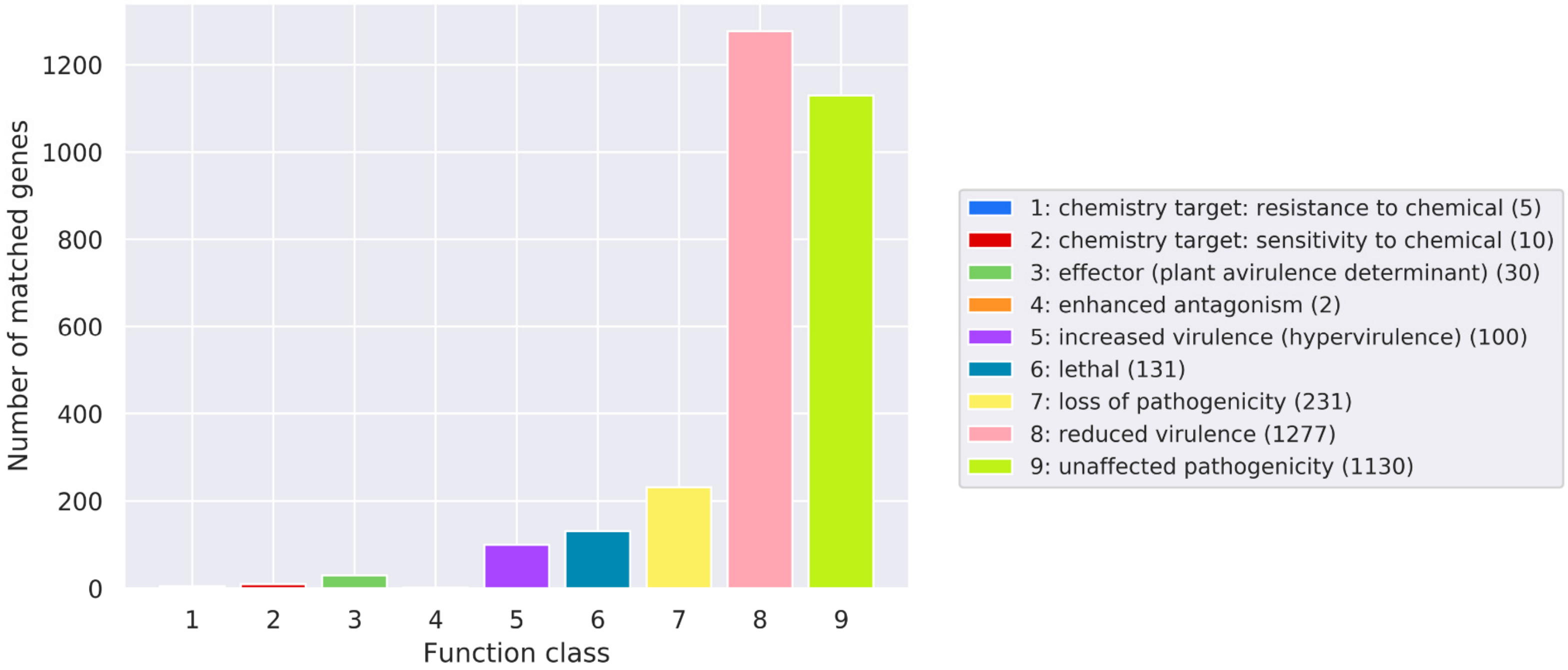

Figure S7. The biological function of functional genes involved in the annotation of *S. yunnanensis* YFCC 1527 in the PHI analysis.

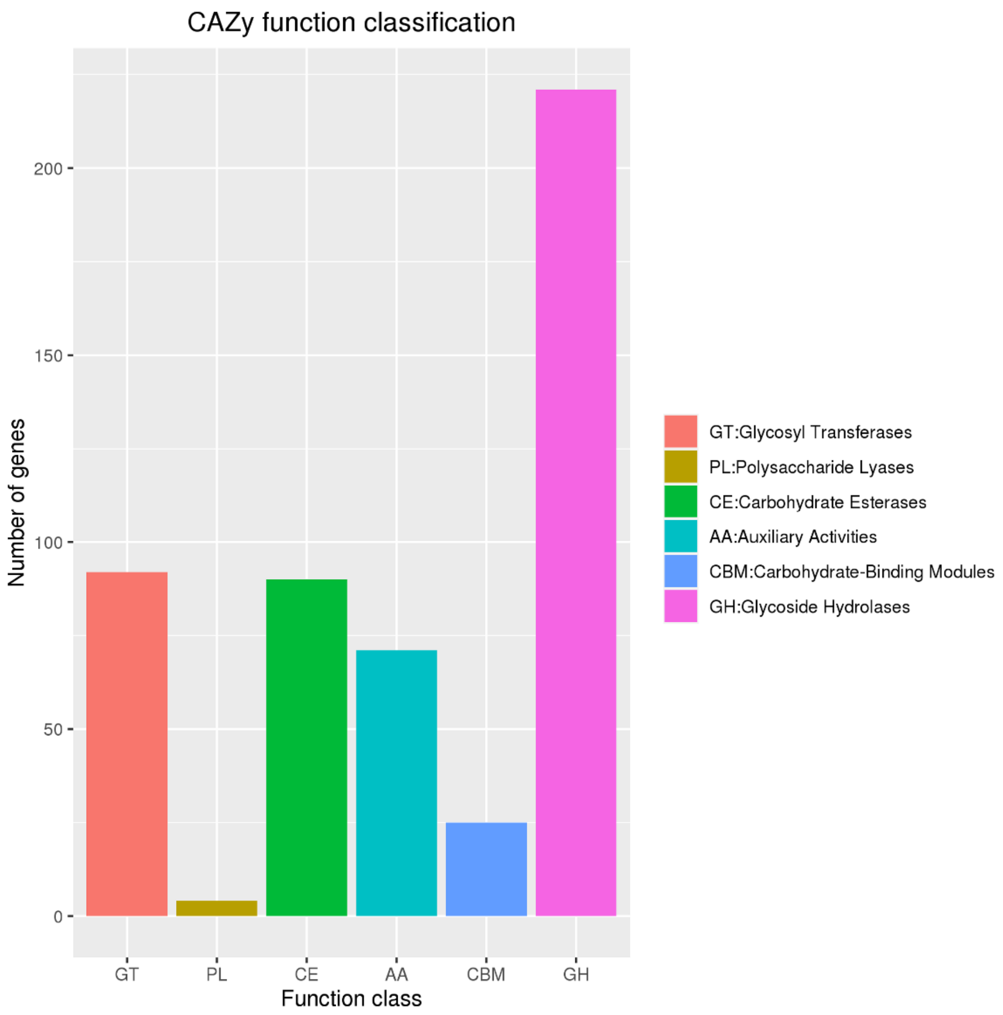

Figure S8. The biological function of functional genes involved in the annotation of *S. yunnanensis* YFCC 1527 in the CAZy analysis.
